# Podcasts in Mental, Physical, or Combined Health Interventions for Adults: Scoping Review

**DOI:** 10.2196/63360

**Published:** 2025-05-07

**Authors:** Elizabeth M Dascombe, Philip J Morgan, Ryan J Drew, Casey P Regan, Gabrielle M Turner-McGrievy, Myles D Young

**Affiliations:** 1 School of Psychological Sciences College of Engineering, Science and Environment The University of Newcastle Callaghan Australia; 2 Centre for Active Living and Learning The University of Newcastle Newcastle Australia; 3 School of Education College of Human and Social Futures The University of Newcastle Callaghan Australia; 4 School of Biomedical Sciences and Pharmacy College of Health, Medicine and Wellbeing The University of Newcastle Callaghan Australia; 5 Department of Health Promotion, Education, and Behaviour University of South Carolina Columbia, SC United States

**Keywords:** podcast, mental health, physical health, health behavior, health promotion, co-design, end user, intervention, scoping review

## Abstract

**Background:**

The increasing prevalence of poor mental and physical health in adults is a global health concern. Given the scope of this problem, scalable and effective treatment interventions are needed. While podcasts (online digital audio files) are becoming more popular, the extent of podcast use in mental, physical, or combined health interventions has not been reviewed.

**Objective:**

This scoping review primarily aims to summarize the available evidence regarding the use of podcasts in health promotion interventions. A series of 5 research questions was designed to systematically review and accurately represent podcast use in current research interventions.

**Methods:**

We conducted a search of electronic databases (MEDLINE, PsycINFO, Embase, CINAHL, Scopus, and CENTRAL), gray literature articles, and relevant journals reported in the English language. Eligible studies targeted adults (aged ≥18 y), included a podcast in at least 1 intervention, and measured a mental or physical health outcome.

**Results:**

Overall, 51 articles (published studies: n=26, 51% and gray literature articles: n=25, 49%) were deemed eligible. In total, 58% (15/26) of the included peer-reviewed studies were published in the last 5 years, suggesting that the use of podcasts as an intervention approach is increasing. On average, 85.6% (n=2104) of the participants included in these research studies were women. In total, 31% (8/26) of the studies contained a female-only sample. In contrast, no research studies contained a male-only sample. Most peer-reviewed published studies (19/26, 73%) and gray literature sources (22/25, 88%) of the podcasts were used within multicomponent interventions, with most targeting physical health outcomes (peer-reviewed publications: 14/26, 54% and gray literature sources: 13/25, 52%). Results pertaining to podcast design, sources, theoretical principles, and thorough process evaluation indicators were heterogeneous.

**Conclusions:**

The versatility that podcasts can offer as a medium for reaching and engaging with participants and end users was evident in this scoping review. While research using podcasts is growing, many (18/26, 69%) studies included in this scoping review were conducted in the United States and sampled female participants, highlighting the need to diversify the field. As expected, there was a high level of variation across the included studies in relation to how podcasts were used and designed within interventions. To address this, a standardized approach would be valuable in guiding researchers and practitioners through both the development and reporting phases of future podcast research, including aspects such as theoretical framework, a description of podcast development (eg, co-design and end-user engagement), objective podcast use, and process evaluation data.

## Introduction

### Background

Globally, the prevalence of people with mental health concerns (eg, anxiety and depression) and physical health concerns (eg, type 2 diabetes and obesity) is increasing [1,2]. Combined, these chronic health conditions account for 74% (approximately 41 million) of all fatalities worldwide every year [3]. Because physical and mental health conditions are interrelated, they share overlapping health consequences that are difficult to address independently of one another [4]. For example, individuals living with a mental health condition (eg, depression and anxiety) are at greater risk of developing other chronic health conditions (eg, sleep disorders, heart disease, and obesity) and vice versa [5,6]. Subsequently, people with a chronic mental health or physical health condition experience a reduced life expectancy of approximately 10 years compared to people without a chronic health condition [7]. Furthermore, the personal impacts of experiencing poor mental and physical health include social isolation, loneliness, relationship problems, and employment challenges [8]. Currently, global estimates suggest that 80% of the people experiencing mental health concerns have limited access to affordable treatment options [1], and 56% of the world’s population is not fully covered by essential health services [9].

Alongside population health approaches, support at an individual level has been shown to help people improve key health behaviors (eg, physical activity, diet, and sleep) and reduce their risk of developing chronic health conditions (eg, diabetes and obesity) [10,11]. Similarly, individual participation in psychotherapy has been shown to improve participants’ mental health by improving emotional and psychological well-being and addressing maladaptive thinking patterns [12]. However, these interventions often have limited scalability (eg, multiple in-person appointments, the requirement for a health professional’s presence during intervention delivery, and financial constraints) [13,14], which limits their reach and may not be appealing for many. These barriers are exacerbated for people living in regional and rural communities due to limited access to health care services (eg, increased travel time, greater geographical spread, and limited availability of health care providers or health care infrastructure) [15]. While the domain of digital health (eg, eHealth and mobile health) interventions has demonstrated potential for improving both physical and mental health [16,17], using these existing digital tools can lead to some challenges [18,19]. These include the need for users to have necessary programs and devices (adaptability), issues with compliance, users being too busy to access services, implementation costs, confidentiality concerns, and the availability of practitioners [18,19]. Subsequently, there remains a need to provide effective individual-level health promotion programs that hold realistic potential for widespread implementation [13,14].

One such opportunity may be the use of podcasts. Podcasts, defined as “online digital audio files that can be played or downloaded to a computer or smart device” [20], present a potentially valuable resource to enhance the scalability of health promotion interventions. While podcasts were once considered the domain of younger population groups [21,22], their use has steadily increased across all age groups [23,24], possibly due to the rise of mobile apps (eg, Spotify), smartphones, and smart devices in cars and homes. From 2008 to 2023, the number of active podcasts increased from 43,000 to >5 million [25]. In 2024, a large multinational study of digital media consumer behavior reported that podcast listenership is at a historic high, with 47% of people (aged >12 y) in the United States listening to a podcast every month and 34% listening every week [26]. Given this widespread global adoption [24,27-31], podcasts may emerge as an effective tool for delivering scalable health promotion programs [13].

As a digital platform, podcasts enable low-cost content distribution while offering listeners flexible and on-demand access to health-related information. Podcasts represent an innovative modality, particularly as they require only basic audio playback capability, eliminating the need for specialized programs or devices (enhanced adaptability). Podcasts can be categorized into 2 types: open-access podcasts and educational podcasts. Open-access podcasts are freely available online and typically feature a more informal, conversational style without adherence to a specific theoretical framework or curriculum [32]. In contrast, educational podcasts are purposefully designed to disseminate information and enhance learning, often focusing on specific courses, skills, or complex topics [33]. Irrespective of the podcast type, the asynchronous and passive nature of podcast listening means content can be played during times when participants might have previously been “too busy” to access other types of support. As such, users can engage in podcast listening while performing other activities, such as commuting, performing household chores, or engaging in physical activity. This functionality enables autonomy among listeners to select where, when, and on which device to play these digital audio files and mitigates the confidentiality concerns of traditional digital health tools. Furthermore, podcasts offer a convenient option for individuals living in rural or remote areas with limited access to health care services or information, allowing them to access mental health support without the need to travel or rely on in-person services. In addition, the low help-seeking behaviors and low uptake of traditional mental health services, particularly among men [34-36], suggest that podcasts may serve as a practical and beneficial alternative, offering accessible and nonstigmatizing resources for individuals who may not otherwise seek help. While podcasts cannot fully replicate practitioner availability or provide tailored support, they can offer consistent, accessible health information and complement existing mental and physical health support.

From a theoretical perspective, multiple foundational theories can help explain the potential efficacy of podcasts in facilitating health-focused information processing, learning, and engagement. User control theory [37] suggests that podcasts can provide listeners with autonomy over their engagement, including timing, location, and the pace at which they wish to listen to each podcast. Cognitive load theory [38] posits that auditory processing, such as podcast listening, demands less mental effort compared to processing other forms of information (eg, written language), thereby preserving the remaining cognitive load for content comprehension. The elaboration likelihood model proposes that individuals actively evaluate and process information through cognitive elaboration, particularly when content holds personal significance, leading to more sustained behavior and attitude change [39]. Research has previously demonstrated that deep-level elaboration of written information promotes enduring behavioral and attitudinal changes more effectively than when information is perceived as irrelevant [39,40]. However, written content is susceptible to selective scanning, which may impede comprehensive information processing [37]. In contrast, while selective attention remains possible, the sequential nature of podcast content delivery eliminates the possibility of content scanning, which may make podcasts a viable tool for people to learn and retain information [41]. Furthermore, regular podcast listeners may develop connections with knowledgeable podcast hosts and acquire new behaviors through imitation, a phenomenon explained by social learning theory [42]. While these theoretical frameworks suggest that podcasts may hold inherent qualities to deliver health-related content and facilitate listener engagement and learning, further examination of these aspects of podcast-based health promotion research is warranted.

Podcasts have the potential to enhance a person’s mental and physical health in many ways. For mental health, podcasts may be used to reduce loneliness (eg, via parasocial relationships and connections) [43], provide psychoeducation to enhance health literacy, and offer evidence-based strategies (eg, skills to increase cognitive flexibility) [44]. For physical health, podcasts may be used to target health behaviors (eg, dietary intake), behavior change techniques, and self-efficacy [41,45]. However, the potential benefits of health-related podcasts must be weighed against certain concerns, particularly regarding information quality. Not all health-related podcasts provide accurate information, and listeners should verify any health advice with qualified health care professionals. Given both the promising potential and notable limitations of podcasts, further research is needed to determine their role and impact as a health promotion tool.

To date, most research into the value of podcasts for disseminating information has been conducted in fields such as education [32,46], kinesiology [47], and medical education [48-51]. For example, podcasts are being used by medical professionals to keep up to date with self-education and learning key content because of their accessibility and portability [48,52]. As such, podcasts are becoming an increasingly dependable educational learning tool for different cohorts of people [32,46-48,52,53]. However, the use of podcasts to deliver health education [54] and mental health support has been limited [44,55]. Of interest, 2 recent studies [44,55] explored the motivations of podcast listeners, specifically exploring mental health podcast listeners’ demographics, motivations, behaviors, and attitudes, to provide insights into why and how people engage with mental health content through podcasting. Evidence across both studies showed that participants were motivated to listen to mental health podcasts to help improve mental health literacy [44,55]. However, to the best of our knowledge, no research, publications, or studies have compiled and summarized the available research assessing podcast use, limitations, and value in both mental and physical health domains.

### Objectives

The use of podcasts as an accessible health intervention tool warrants rigorous examination, particularly given their potential to impact both physical and mental well-being. However, there remains a critical gap in systematically understanding how podcasts specifically function as health promotion and support tools. This gap needs to be addressed through a comprehensive literature review of podcast-based studies to help establish the empirical foundation for the use of podcasts in health research. As such, the objective of this scoping review was to conduct an exploratory search to review and summarize the use of podcasts in mental, physical, or combined health interventions in adults. Specifically, this review investigated the following research questions:

1. How many studies targeting mental, physical, or combined health outcomes in adult populations have used podcasts in at least 1 study arm?
2. What health outcomes were targeted by the studies?
3. How have podcasts been used within the interventions?
4. How were the podcasts designed (eg, structure, format, theoretical framework, topics, and end-user involvement)?
5. What process evaluation data were reported regarding podcast use (eg, barriers and facilitators)?

## Methods

### Overview

The scoping review followed the Arksey and O’Malley [56] framework stages (identify the research question; identify relevant studies; select studies; chart the data; and collate, summarize, and report the results). The Joanna Briggs Institute methodology guidance for scoping reviews and the PRISMA-ScR (Preferred Reporting Items for Systematic Reviews and Meta-Analyses Extension for Scoping Reviews) checklist [57,58] were followed to maximize the methodological rigor of this review (Multimedia Appendix 1). The protocol for this scoping review was registered prospectively with Open Science Framework [59].

### Identification of the Research Questions

The research objectives of this scoping review were designed to be wide ranging and comprehensive to capture the full breadth of available evidence [56]. The broad scope of the examination allowed for a thorough investigation; however, it was likely to be diverse and heterogeneous in nature (eg, types of outcomes and reasons for podcast use). As a result, the intent to focus on capturing and mapping the broad content was important, with less of a focus on efficacy.

### Identification of Relevant Studies

This scoping review followed a systematic literature search that was guided by the recommended population, concept, and context (PCC) framework [57,58]. Specifically, the criteria in the subsequent sections were used to identify relevant articles.

#### Population

The target population was adults aged ≥18 years. Family-focused studies (eg, comprising both adults and children) were eligible for inclusion if the study reported at least 1 mental or physical health outcome in the adult participants.

#### Concept

This scoping review was conceptualized in 2 ways, namely interventions incorporating podcasts and physical, mental, or combined health interventions.

##### Interventions Incorporating Podcasts

For inclusion, studies were required to have featured a podcast within at least 1 study arm, either as the sole intervention component or within a multicomponent intervention. For the purpose of this scoping review, podcasts were operationalized as online digital, audio-only recordings of a radio broadcast or similar program made available on the internet for downloading to a personal audio player (adapted from the Oxford [20] definition of podcasts). In line with previous literature, video-based podcasts, webcasts (ie, video-only content hosted on a website), or other educational materials that were primarily web based were not considered eligible for the review [49].

##### Physical, Mental, or Combined Health Interventions

To be considered eligible for inclusion, studies needed to test at least 1 intervention designed to improve physical health or mental health, which may include education or behavior change techniques (eg, self-monitoring of behavior and goal setting related to dietary intake). However, studies also needed to report changes in at least 1 mental health outcome (eg, depression, anxiety, and quality of life [QOL]) or physical health outcome (eg, weight and blood pressure). Studies were not eligible if they used podcasts to focus on specific mechanistic tasks with no clear intent to initiate lasting mental or physical health changes (eg, listening to a metronome sound via a podcast, which is designed to improve step cadence while walking). Studies that focused on patient education to improve knowledge alone (without reporting changes in a specific physical or mental health outcome) were excluded.

The complete eligibility checklist is provided in Multimedia Appendix 2 [1,60-62].

#### Context

Inclusion criteria encompassed English-language publications with human participants as the target population. This review considered articles published in peer-reviewed journals and sources of gray literature (operationalized as conference abstracts, dissertations, published protocols, or clinical trials that were registered but not yet published). Given the potentially dynamic nature of this research area, gray literature sources were included to provide a more comprehensive overview of the field and capture emerging findings that may not yet appear in peer-reviewed publications (which can often be delayed while going through the publication process). No date restrictions were applied to the search.

#### Search Strategy

In June 2023, a single author (EMD) applied the search strategy in MEDLINE, PsycINFO, Embase, CINAHL, Scopus, and Cochrane Library (CENTRAL) electronic databases. To be retrieved in the primary search, articles needed to include the term “podcast” anywhere in the text of the article *and* at least 1 term from a “study design” group (eg, intervention OR program OR trial) in the title, abstract, or keywords. Multimedia Appendix 3) provides search syntax and results for all databases. Where needed, the search strategy was adapted for each database, including keywords and index terms.

In addition, a series of secondary search strategies was applied in January 2024. The first reviewer (EMD) conducted a search of the reference lists for all eligible articles and a search for all articles that had cited these articles according to Google Scholar. Separately, searches to identify sources of gray literature were also conducted. These included conference abstracts, dissertations, and theses that were identified in the primary database search as well as a comprehensive search of 3 clinical trial registries (Australian New Zealand Clinical Trials Registry, United States of America Clinical Trials Registry, and World Health Organization International Clinical Trials Registry Platform). Clinical trial registries were searched for any completed, ongoing, or unpublished trials that included podcast* in the registration form.

### Selection of Eligible Studies

All articles identified from the initial database search were exported using Endnote (reference management software, version 20; Clarivate). Duplicates were manually reviewed and removed where necessary after an initial automated detection process. These were then transferred from Endnote to Covidence (Veritas Health Innovation Ltd) for screening, with subsequent duplicates also identified and removed via the in-built process. The lead author (EMD) conducted the title and abstract screening process. At the commencement of full-text screening, the first reviewer (EMD) and a second author (MDY) independently coded 10.6% (17/160) of the articles to ensure that there was agreement among the reviewers (absolute agreement=94%). With a high level of agreement between the reviewers, the first reviewer (EMD) completed the coding of the remaining articles. Reasons for exclusion were recorded at the full-text screening stage and reported in the PRISMA (Preferred Reporting Items for Systematic Reviews and Meta-Analyses) flowchart.

### Charting the Data

The first reviewer (EMD) developed the data extraction template in Covidence, which was refined after discussion with the review team (MDY and CPR; Multimedia Appendix 4). Key data items of interest were selected based on the PCC framework [57], which included study characteristics (eg, author), participant data (eg, targeted demographics of the sample population), and outcome measures, with a particular focus on podcast development, design, and use. Moreover, podcast use data were extracted if reported, along with process evaluation insights (eg, facilitators and barriers) in relation to podcast engagement. To test the coding form, 5 peer-reviewed published articles were independently double coded by 2 reviewers (EMD and CPR). Following this, EMD, CPR, and MDY met and reviewed the data. Any disagreements were resolved through discussion. As disagreements occurred infrequently, the reviewers EMD and CPR proceeded with double coding of the remaining peer-reviewed published articles. Gray literature articles were single coded by the lead author (EMD) due to the heterogeneity of the literature identified. After all data were extracted, the corresponding author from each article (both peer-reviewed published studies and sources of gray literature) was contacted via email to verify whether the data were correct and provide additional missing information. A total of 53 emails were sent to corresponding authors, and 22 responses were received.

## Results

### Overview

**Figure 1:**
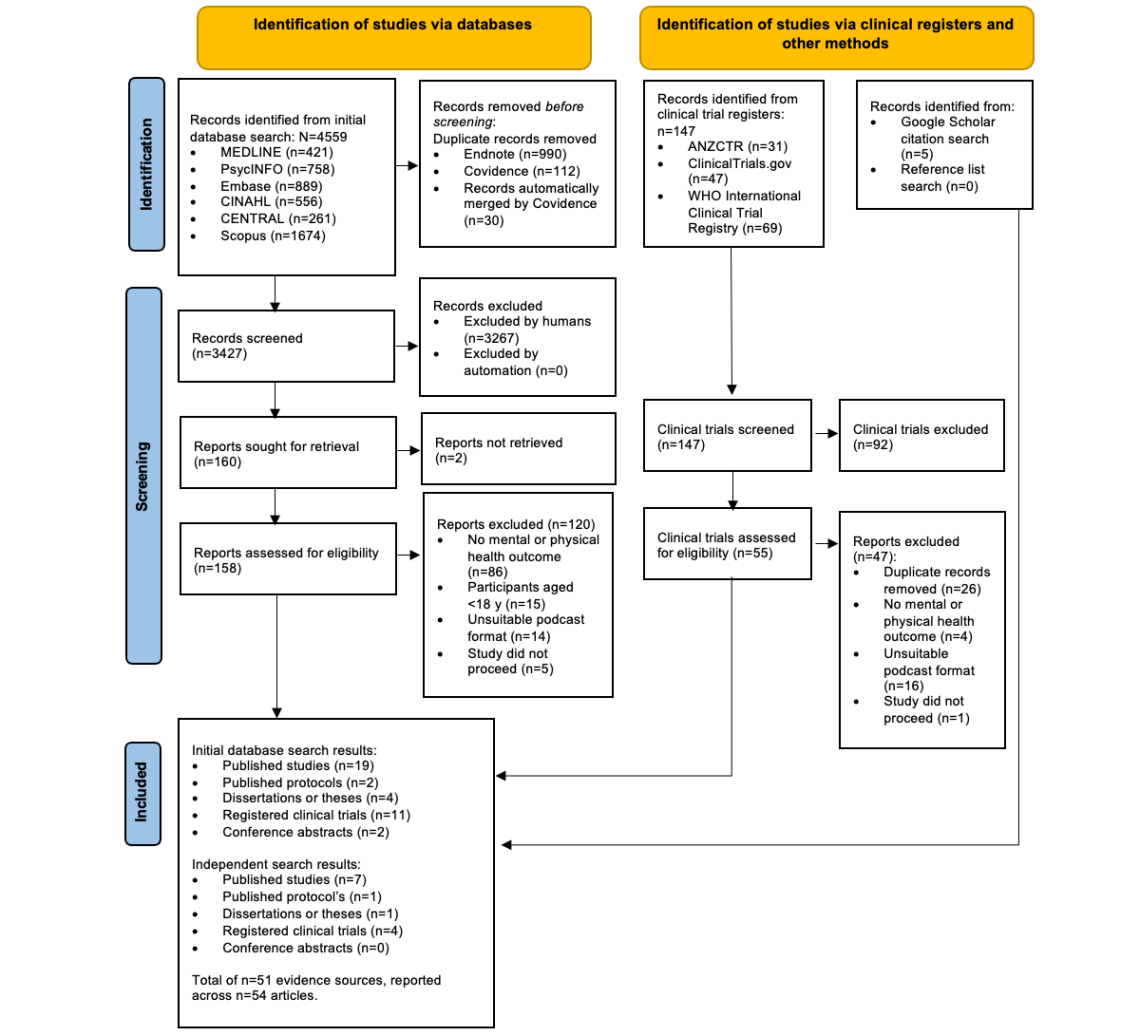
PRISMA-ScR flowchart of studies through scoping review process. ANZCTR: Australian New Zealand Clinical Trials Registry; WHO: World Health Organization.

Figure 1 depicts the flow of articles identified and included in this scoping review. The selection process is described and presented using the PRISMA-ScR flowchart [57,58]. The data are presented in a tabular form and accompanied by a narrative summary of the results in relation to the objectives of the scoping review.

In total, there were 51 evidence sources reported across 54 articles eligible for inclusion within this scoping review [41,45,63-114]. Overall, 26 (51%) independent peer-reviewed published studies (consisting of 29 reports) and 25 (49%) sources of gray literature were included in this scoping review.

The initial database search returned 3457 unique references after duplicates were removed, and 30 references were automatically merged with the primary reference for the trial. Following this, 3427 evidence sources were screened for title and abstract (n=3267, 95.33% were excluded). The remaining 160 (4.67%) evidence sources were assessed against the PCC framework for full-text eligibility. This process identified 21 peer-reviewed published studies (from 19 unique studies) [41,45,66-68,70,73,77,80,83,86-90,101,103,107,108,110,113], 11 registered (but unpublished) clinical trials [63,65,78,81,82,85,97-99,106,112], 4 PhD dissertations or theses [69,72,74,84], 2 published protocols [79,109], and 2 conference abstracts [94,111] that were eligible for inclusion. Following this, a manual citation search using Google Scholar was conducted for the 19 peer-reviewed published studies, which identified a further 10 evidence sources, including 4 peer-reviewed published studies (consisting of 5 reports) [75,92,100,102,104] and 1 dissertation [93] suitable for inclusion into the scoping review. Following this, a manual reference list search of the 19 peer-reviewed published studies was also carried out; however, it did not return any new evidence sources. Separately, examination of gray literature sources identified 3 registered clinical trials that were later published as peer-reviewed studies [64,71,105], 4 ongoing registered clinical trials [76,91,95,96], and 1 published clinical trial protocol [114], all of which were eligible for inclusion.

### How Many Studies Targeting Mental, Physical, or Combined Health Outcomes in Adult Populations Have Used Podcasts in at Least 1 Study Arm?

Multimedia Appendix 5 outlines all the key characteristics of included evidence sources.

#### Peer-Reviewed Published Studies

Of the 26 included peer-reviewed published studies, most have been conducted in the United States (n=19, 73%), with the remainder distributed across Australia (n=3, 12%), Iran (n=2, 8%), Sweden (n=1, 4%) and Canada (n=1, 4%; Multimedia Appendix 5). Female participants constituted an average of 85.6% (n=2104) of the total study population. A total of 8 (31%) studies comprised female-only samples [64,68,75,87-90,92,100,102]. In contrast, no research studies consisted of male-only cohorts. Although participant groups were diverse (eg, adults who were overweight or obese, working mothers and pregnant women, patients with cancer, university students, grocery shoppers, veterans, military spouses, and patients with various health conditions), the limited number of studies included in this review did constrain the strength of these results and ability to draw robust findings. The sample sizes across all studies demonstrated considerable variation, ranging from 12 to 351 participants. The age distribution of participants spanned from young adulthood (aged ≥18 y) to older adults (aged up to 90 y), with the mean age of participants most commonly between their late 20s to mid-50s. Methodologically, the included studies primarily used randomized controlled trial designs. The assessment periods varied substantially, ranging from immediate posttest intervention evaluations to longitudinal follow-ups (12 months after baseline). Of the total peer-reviewed studies analyzed, 15 (58%) studies were published between 2019 and 2024.

#### Gray Literature

The 25 sources of the gray literature followed a similar representation of countries as the peer-reviewed published papers, with 15 (60%) conducted in the United States; 4 (16%) in Australia; 3 (12%) in Iran; and 1 each from Columbia (4%), Germany (4%), and Portugal (4%). Participant samples were predominantly female, ranging from 60% to 90% across studies, and age distributions spanned from 18 to 65 years.

Interventions focused on a heterogeneous array of health domains and participant populations, including pregnant women, cancer survivors and patients with cancer, caregivers, people with chronic conditions (epilepsy, fatty liver disease, and celiac disease), adults who were overweight or obese, mental health focus groups, active-duty service members, and paramedic support network members. Sample sizes demonstrated considerable variability, ranging from 15 to 2297 participants, with most (10/15, 67%) studies targeting cohorts of 50 to 350 participants. Methodologically, the identified sources of gray literature predominantly focused on specific health populations with well-defined intervention goals.

### What Health Outcomes Were Targeted by the Studies?

Multimedia Appendix 5 outlines all the key characteristics of the included evidence sources.

#### Peer-Reviewed Published Studies

Of the 26 included peer-reviewed published studies, 14 (54%) targeted physical health only [41,45,64,66-68,73,75,77,87,88,92,102,104,107,108,113], 8 (31%) targeted mental health only [70,71,80,83,86,101,103,110], and 4 (15%) targeted both physical and mental health [89,90,100,105]. Weight loss emerged as the predominant outcome measure [41,45,73,77,87,88,107,108]. QOL was the second most frequently assessed outcome, measured in 2 distinct ways: health-related QOL, which specifically examined health impacts (12-Item or 36-Item Short Form Survey) [64,100], and general QOL, which took a broader view incorporating both individual factors and perspectives and social and cultural influences on well-being (World Health Organization Quality of Life Brief version) [105].

#### Gray Literature

Among the 25 sources of the gray literature, 13 (52%) targeted physical health only [69,79,81,85,93-97,99,106,109,111], 8 (32%) targeted mental health only [63,72,74,76,78,84,112,114], and 4 (16%) focused on both physical and mental health [65,82,91,98].

### How Have Podcasts Been Used Within the Interventions?

Information that describes the key characteristics of included evidence sources and specific podcast characteristics are provided in Multimedia Appendix 5 and Multimedia Appendix 6.

#### Peer-Reviewed Published Studies

Of the 26 peer-reviewed published studies, only 7 (27%) conducted a podcast-only intervention [66-68,75,80,92,101,102]. The remaining 19 (73%) studies [41,45,64,70,71,73,77,83,86,88-90,100,103-105,107,108,110] used podcasts within a multicomponent intervention (Multimedia Appendix 5). Podcasts were often used among other intervention components, including exercises to monitor progress (eg, dietary and physical activity intake) [41,45,73,77,107,108], access to online resources [64,89,90,100,103,110], phone or online consultations with health care practitioners [64,87,88,100,113], and social support (eg, text message or group activities with other participants in the study) [45,89,90,103]. Only 2 (8%) studies assessed the podcast component as a primary intervention approach [75,80,102]. In total, 14 (54%) peer-reviewed studies reported using preexisting podcast content (eg, “Weight Loss And The Mind 2.0” [41]) in their studies [41,64,70,71,75,80,83,86-89,92,101,102,104,110,113]. The range of preexisting podcasts came from a variety of resources, including publicly available podcast channels, such as Spotify and Podbean. Many of these podcasts (14/26, 54%) had been selected as they were considered by researchers to be related to a key aspect of the study; however, often provided limited information detailing the podcast selection process. By comparison, 42% (11/26) of the studies included original, researcher-developed content [41,45,66-68,73,77,87,88,90,107,108,113]. Notably, theory-based podcasts developed in 2009 [41] and 2011 [45] designed to target weight loss in adults had been replicated and adapted to other sample populations (eg, pregnant women who were overweight and obese) in several (4/26, 15%) of these studies [73,77,87,88,108,113]. Moreover, 8% (2/26) of the studies [41,87,88,113] compared a researcher-developed podcast (in the intervention condition) to a preexisting podcast (in the comparator or control condition), citing that the podcasts in both conditions had been matched in frequency and duration.

#### Gray Literature

In total, 12% (3/25) of the gray literature sources outlined a podcast-only intervention [74,84,112], with 88% (22/25) embedding podcasts within a multicomponent intervention [63,65,69,72,76,78,79,81,82,85,91,93-99,106,109,111,114].

Across all 25 sources of gray literature, podcasts were most used within intervention conditions (n=20, 80%) [63,65,72,74,76,78,81,82,84,85,91,93-99,111,112], with 3 (12%) sources including podcasts in both intervention and comparator or control conditions [69,106,109], and only 2 (8%) in comparator or control condition [79,114]. Researcher-developed podcasts were used in 7 (28%) studies [63,69,82,84,93,106,109], while preexisting podcasts were used as a comparison or control condition in 4 (16%) studies [65,69,74,114]. The remaining (15/25, 60%) sources of gray literature did not report this level of detail.

### How Were the Podcasts Designed (Structure, Format, Topics, and End-User Involvement)?

Multimedia Appendix 6 describes the podcast design, format, and topics covered in the included articles. The reported results varied widely between all studies.

#### Peer-Reviewed Published Studies

##### Podcast Design Including the Source, Presenters, Number, Frequency, and Duration of Podcasts)

Very few (2/26, 8%) studies provided detailed information regarding the overall podcast design. The paucity of information concerning structural elements, including host or presenter composition, was reported in only 15% (4/26) of the studies [41,68,87,88,113] and presented a significant barrier to identifying active components and mechanisms of effective podcast designs. Some preliminary details, such as the name or links to preexisting podcast information, were also noted by 38% (10/26) of the studies [41,68,70,71,75,83,92,101,102,104]. Podcasts were most frequently scheduled for release to participants using a staggered style, once or twice per week [41,45,73,77,87-90,105,107,108,110,113]. The number of podcasts included in each study arm ranged from 1 to 48, with an average of 18. Where reported, with the exception of 4% (1/26) of the studies [105], the duration of all podcasts was <30 minutes per podcast or episode.

##### Theoretical Framework and End-User Engagement

Theoretical frameworks or behavior change principles were explicitly documented in 54% (14/26) of the included studies [41,45,64,66,67,73,77,89,90,101,103,107,108,110]. Social cognitive theory [115] emerged as the predominant theoretical foundation, implemented in 27% (7/26) of the interventions [41,45,64,73,77,87,88,108,113], followed by self-determination theory [116] cited in 2 (8%) studies [89,90]. Notably, only 12% (3/26) of the studies [41,92,107] specifically included a theoretical rationale to explain why podcasting may be an effective means of delivery and learning health-related information, explicitly outlining how the use of podcasts in these interventions aligns with key concepts from information processing theories, such as user control theory, cognitive load theory, and the elaboration likelihood model. However, many other researchers often described theoretical frameworks or principles that related to the entire intervention rather than drilling down to provide a rationale for the use of podcasts.

Limited implementation of participatory research methods was observed, with only 12% (3/26) of the studies documenting end-user engagement or co-design processes incorporating stakeholder input (eg, including individuals with lived experience or health care professionals) in the intervention development phase [68,90,101].

##### Topics

Despite the brevity of details to describe podcast design and format, many (23/26, 88%) studies did provide a brief outline of topics covered in the podcasts [41,45,66-68,70,71,73,75,77,80,83,86,88-90,92,101,104,105,107,108,110]. As shown in Multimedia Appendix 6, podcast topics that related to the overarching intervention message or outcome were developed or selected. The topics were varied, with the most common being podcasts that focused on weight loss, diet or nutrition, and well-being, often incorporating topics that covered physical health, mental health, and health behavior changes (eg, stress, weight loss, physical activity, overcoming barriers, and goal setting). The most widely used podcasts were those that had been developed by Turner-McGrievy et al [41,45]. The theory-based podcasts were developed to reinforce key intervention messaging relating to weight loss, diet, and exercise, using a soap opera storyline throughout the podcast series to engage participants [41,45].

#### Gray Literature

##### Podcast Design Including the Source, Presenters, Number, Frequency, and Duration of Podcasts

Limited information to describe podcast design was reported within the sources of gray literature. Details outlining podcast presenter information were reported by only 12% (3/25) of the studies [65,82,84]. Likewise, information describing the number, frequency, and duration of podcasts within each intervention arm was often not reported. However, from the included details, the number of podcasts varied (range 2-69 podcast episodes), with many (8/10, 80%) studies including up to 10 podcast episodes in their intervention [65,69,74,82,84,95,96,112]. Commonly, the duration of podcasts was between 10 and 25 minutes [63,69,74,84,95,96]. Yet, the frequency in which podcasts were made available to participants was inconsistent, varying from podcasts being released to participants all at once [69,74] to once or twice per month [95,96,109].

##### Theoretical Framework or Foundational Principles and End-User Engagement in Design

Collectively, 28% (7/25) of the gray literature sources noted any information outlining theoretical underpinnings or foundational principles [65,69,74,84,93,109,112]. Social cognitive theory [115] was the most (4/25, 16%) commonly noted theory [65,69,93,109]. Only 1 (4%) registered clinical trial noted plans for a community advisory board to be involved in the selection of podcast content and topics [82].

##### Topics

In total, 48% (12/25) of the gray literature sources included information regarding podcast topics [63,65,69,74,79,81,82,84,93,109,112,114]. Podcast topics aligned to the overarching theme of the proposed intervention, such as health or behavior change information, to reinforce key learning for participants (eg, well-being and stress).

### What Process Evaluation Data Were Reported Regarding Podcast Use (Barriers and Facilitators)?

Process evaluation data are briefly described subsequently and reported in Multimedia Appendix 7. Given the scarcity of process evaluation data provided in the gray literature sources, this section is focused only on peer-reviewed published studies.

#### Data Use

Of the 26 published studies, 9 (35%) studies objectively measured podcast use (eg, podcast downloads) [41,45,73,77,80,87,88,107,108,110,113], and 5 (19%) studies used subjective measures (eg, participants’ self-reported podcast listenership) [68,75,89,92,101,102]. A total of 8 (31%) studies did not report any podcast use data [64,66,67,86,90,100,103,105]. In 4 (15%) studies, podcasts were queued to play for participants, or participants were issued instructions and monitored by researchers to ensure that podcast content was listened to [70,71,83,104].

#### Facilitators

A range of facilitators was identified across 46% (12/26) of the studies [41,66,68,73,75,77,89,90,92,101,102,108,110]. From a practical aspect, podcasts were reported to be useful and innovative [89,90,101], convenient and easy to use [68,90], low cost [41,66], and an easy method to disseminate information for researchers [41,66]. Participants reported enjoying podcasts that increased or enhanced their knowledge of topics that they found to be of interest [41,66,75,90,92,102]. Other (4/26, 15%) studies reported that participants liked podcasts that were short and succinct [66-68,89]. Some (3/25, 12%) studies also provided training at the baseline assessment, supplied user guides detailing how to access and listen to podcasts [73,108,110], or provided links to podcast scripts for participants to revisit and read [68].

#### Barriers

Reported barriers were categorized into concerns relating to the format of the podcast, access and technical issues, and participant attrition. Participants in 31% (8/26) of the studies reported concerns with the podcast content or format [41,66,75,80,87-90,92,102,113]. For example, some participants reported that they did not resonate with the podcast content or felt it to be unrelated to their situation [75,80,87,88,92,102], while participants from other studies recognized more practical challenges. Podcast duration was also identified as a barrier, particularly when key content was repeated in other intervention components (handouts, phone calls, and workbooks) [87-90]. Furthermore, barriers due to accessibility and technical issues were reported in several (10/26, 39%) studies [66,74,77,80,87,90,92,101,108,110]. These included difficulties associated with podcast sound or hearing quality [66,80], internet connectivity, or platform functionality [77,80,90,101,108]. An unexplained increase in participant attrition in the second half of podcast interventions was another recognized obstacle [87,88,92]. Moreover, some researchers reported difficulties with the podcast platform failing to capture podcast listening time [87,88,92].

#### Other Perceptions of Podcasts

A range of other process evaluation data reported across the studies is summarized in Multimedia Appendix 7. For example, some researchers provided evaluation information by describing listening habits, such as listening locations (eg, car, home, and work) or devices used to listen to the podcast (eg, smartphone and computer) [41,101], while other researchers developed Likert scales to measure the perceived acceptability of podcasts [66,68,80,87-90,101].

## Discussion

### Principal Findings

The primary aim of this scoping review was to examine and summarize the use of podcasts in mental, physical, or combined health interventions targeting adults. Following a systematic review process, we detected 51 eligible sources (published studies: n=26, 51% and gray literature sources: n=25, 49%). Many (18/26, 69%) of the studies were conducted in the United States and sampled female participants. As expected, there was a very high level of variation across the included studies in relation to how podcasts were used and designed within the interventions. Moreover, key aspects of the interventions, such as co-design participation, end-user engagement, and thorough process evaluation, were lacking. Despite this, it was apparent that podcast use in empirical research has grown in recent years, with this medium possessing great utility and versatility to reach and engage participants in health promotion research.

Notably, most (15/26, 58%) of the studies included in this review were published in the last 5 years. Since the initial podcast-based studies emerged in 2009, the recent increase in podcast use in health research represents an emerging area of scholarly interest. These observed trends align with data derived from multinational surveys examining digital media consumption behavior [21,22,24,26], which indicate that approximately two-thirds of individuals in the United States regularly engage with podcasts (defined as listening to at least 1 podcast monthly) [26]. This synthesis of information reveals a notable lack of widespread representation across countries and sample populations within the literature. Specifically, there was limited demographic diversity across study populations, with research predominantly conducted in the United States, primarily recruiting female participants and focusing on body weight interventions. Moreover, only 27% (7/26) of the published studies used podcast-only interventions, which significantly constrains the generalizability of findings. In addition, many (14/26, 54%) studies included in this scoping review used preexisting podcasts, with this reliance on existing content preventing a thorough evaluation of podcast development processes or their alignment with evidence-based frameworks. While podcasts demonstrate potential as an engagement tool for participants, further research is essential to establish their effectiveness and enhance their generalizability across diverse contexts and settings.

The analyzed studies showed substantial variation in how podcast design elements and critical characteristics were documented, including the rationale for podcast selection and presenter demographics. This inconsistent reporting of podcast-specific variables has also been noted in adjacent fields [33,47], and it creates challenges for both replication studies and systematic evaluation of design features that may influence intervention outcomes. While podcast topics and episode duration (predominantly <30 min) were consistently reported, documentation of design elements varied significantly across studies. For example, researchers using preexisting podcasts rarely provided sufficient detail to support their podcast selection or quality assessment processes. These omissions limit the generalizability of both the podcast and the content. Similar to other digital health fields, while some mental health podcasts can be beneficial, inadequate quality assessment introduces potential risks of harm [117-119]. Comprehensive reporting of presenter characteristics, including demographics, professional credentials, and relevant lived experience, is also crucial for evaluating critical expertise, credibility, and potential sources of bias [120]. As podcasts become increasingly integrated into health promotion research, researchers must provide more transparent information about podcast design and presenter profiles to help listeners assess information credibility and reliability [120].

The implementation and documentation of theoretical frameworks in podcast design demonstrated notable disparities across studies. While some researchers provided robust theoretical frameworks, others omitted this methodological component completely. The integration of theoretical frameworks may strengthen intervention implementation and evaluation rigor, potentially optimizing outcomes and goals of the study (eg, building autonomy or self-efficacy among participants) [121]. These concerns align with similar methodological limitations in podcast design across other disciplines, including kinesiology and education, and, as a result, have impacted the ability of future researchers to develop evidenced-based study protocols and high-quality podcast creation [47,53]. The implementation of standardized reporting protocols for theoretical design elements would significantly benefit future health promotion researchers using podcast-based interventions.

Importantly, while research co-design and end-user engagement are becoming key features of health promotion interventions [122], there was a stark lack of end-user engagement described and thoroughly evaluated in the design of the podcast interventions included in this scoping review. Studies that adopt a circularity co-design approach by fostering genuine end-user engagement during development and implementation have shown promise in other mobile health reviews [123,124]. This type of input has helped to uncover unexpected participant insights [123] and may help to address some of the identified barriers (eg, perceived lack of relevance and relatedness to users’ needs) reported in this scoping review. Such participatory methods bring about the opportunity to engage and potentially reduce inequities in underrepresented populations [124], helping to overcome barriers by aligning content to the unique needs and preferences of the end user. Collectively, these insights suggest that co-design and end-user engagement are valuable and should be used to inform future podcast health promotion research.

Many (14/26, 54%) of the published studies captured process evaluation data, although diverse approaches were used, which highlights another missed opportunity. Process evaluations are an important consideration within an intervention as they can assist researchers in better understanding the associations between podcast components [125], in particular, why the intervention may or may not have worked, and foster better engagement with end users [125]. While it was difficult to summarize findings from this scoping review, some key insights were identified. Encouragingly, podcasts were described as innovative and useful, especially when aligned with relatable topics that enhance knowledge or pique personal interests. Participants also liked the anonymity, ease of access, and convenience of being able to listen to podcasts at a time that suited them. Correspondingly, these themes align with participants’ perceptions of the benefits of other adjacent digital health interventions, such as digital mental health promotion interventions [126,127], lifestyle behavior change apps [128], and eHealth services [19]. The difficulties included inaccessibility and technical concerns (eg, poor sound quality) or poor digital and technical literacy levels of the participants (eg, unable to navigate or listen to podcasts) and align with some of the top barriers identified in related literature [19,128,129]. Furthermore, while some efforts were made to capture the objective (eg, podcast downloads or listen time) and subjective (eg, self-report) podcast use data by studies included in this scoping review, the nonstandardized methods make the results difficult to generalize. In line with comparable intervention approaches, recording objective use data is recognized as an important method to highlight the relative use [130] and, along with a wide variety of process evaluation indicators, should be used to identify and guide the quality intervention implementation in future podcast-based interventions.

### Strengths and Limitations

This scoping review contained several strengths and limitations. First, we implemented a systematic and comprehensive search strategy across several databases and sources of gray literature to provide a more thorough snapshot of this rapidly emerging field. Second, this scoping review focused on interventions that targeted both mental health and physical health, which also aligns with recognized research, highlighting that the 2 health domains are interrelated [4]. Third, we followed a thorough data extraction process with 2 coders for each eligible article. However, our study had some notable limitations. We restricted our analysis to English-language studies that explicitly used the term “podcast” in their text, which may have inadvertently excluded relevant research that met the conceptual definition of a podcast intervention but did not use that specific terminology. For feasibility reasons, the initial screening of titles and abstracts was conducted by a single reviewer (EMD), which could have potentially introduced some screening bias or errors. Given the emerging nature of podcast interventions in mental and physical health, our research team opted to provide a descriptive summary of the available data rather than attempting a comprehensive effectiveness synthesis. Finally, given the very broad focus of our review (on any physical or mental health outcome), we did not include qualitative studies or those that reported on changes in health knowledge alone. These would be valuable areas for future research to explore.

### Recommendation for Future Research

This scoping review has identified several research gaps and provided some valuable recommendations for health promotion researchers using podcasts (refer to Textbox 1 for a summary). To advance the field, standardized methodological frameworks and recommendations are essential to guide researchers and practitioners through both the development and reporting phases [131]. Such frameworks should, at a minimum, encompass (1) a comprehensive theoretical foundation and an explicit rationale that underpins podcast design and use, (2) systematic documentation of podcast development methodology, and (3) quantitative analysis of podcast engagement metrics and use data.

**Summary of recommendations for future research.**

- Test podcast use in diverse populations samples
- Incorporate justification and rationale for podcast selection and sources
- Describe podcast design and key characteristics, including theoretical framework, number and duration of episodes, and topics
- Illustrate podcast presenter information (eg, demographic details, professional qualifications, and personal background or lived experience)
- Consider co-design in the design phase
- Report podcast use data—objective indicators (eg, download or time spent listening)
- Report podcast process evaluation data (eg, facilitators and barriers to use)

Importantly, addressing the lack of co-design and end-user engagement as well as the collection of thorough evaluation process data remains a pivotal need that warrants attention in future studies. This methodological rigor would facilitate reproducibility, enable meaningful comparative analysis that can identify and overcome key barriers and facilitators to podcast use, and strengthen the empirical basis for podcast-based interventions [122,131,132]. To diversify the field, future studies should include greater representation of diverse populations and participant samples (eg, the inclusion of more male participants in studies or people living in remote and isolated regions) across different countries and in a broad range of topics. Research investigating the rising number of mental health and physical health interventions has shown that podcasts may enhance physical and mental health outcomes. As such, a future systematic review and meta-analysis to summarize results and provide further practical recommendations for use beyond research is warranted when sufficient data are available. Moreover, as the adoption of health promotion podcasts continues to expand in research, the need to develop a rigorous conceptual framework for systematic podcast evaluation should be considered. This methodological approach parallels existing evaluation frameworks in other digital health domains, such as mobile health apps [133].

### Conclusions

Year over year, the number of podcasts across the globe continues to rise [24,26-30]. To the best of our knowledge, this is the first scoping review to comprehensively investigate podcast use in mental, physical, or combined health interventions. The results summarized in this review provide a valuable initial step in exploring and understanding the many ways that podcasts can be used with physical and mental health interventions. As the listenership of and number of podcasts continue to grow worldwide, they serve as a medium with great potential and are an important consideration for health promotion researchers as a novel method to reach and engage participants requiring health behavior change.

## Appendix

Multimedia Appendix 1 PRISMA-ScR checklist.

Multimedia Appendix 2 Eligibility checklist.

Multimedia Appendix 3 Syntax search strategy.

Multimedia Appendix 4 Data extraction template.

Multimedia Appendix 5 Key characteristics of included evidence sources.

Multimedia Appendix 6 Podcast characteristics.

Multimedia Appendix 7 Podcast use data, process evaluation data, and related information.

## Abbreviations

PCC: population, concept, and context
PRISMA: Preferred Reporting Items for Systematic Reviews and Meta-Analyses
PRISMA-ScR: Preferred Reporting Items for Systematic Reviews and Meta-Analyses Extension for Scoping Reviews
QOL: quality of life
