# Supplementary material for: Podcasts in Mental, Physical, or Combined Health Interventions for Adults: Scoping Review
Source: J Med Internet Res. 2025 May 7;27:e63360. doi: 10.2196/63360 (PMC12096026; doi:10.2196/63360)
Supplement: Multimedia Appendix 2 [file jmir_v27i1e63360_app2.docx]

**Supplementary material (S2):**

**Podcasts in Mental and/or Physical Health Interventions for Adults: A Scoping Review**

**Study eligibility checklist**

**General inclusion criteria:**

- Study article published in an electronic database, grey literature or peer-reviewed journal.
- English language

**Participants:**

- Adults 18 years and older
- *Interventions that focus on families (e.g., adult and children participants), are eligible for inclusion if the study includes a podcast component and at least one mental and/or physical health outcome measure in adults.*
- *Studies that recruited participants aged 17 years or younger were not eligible.*

**Intervention:**

- Studies had to include a podcast component within the intervention (e.g., either multi-component or podcast-only). Podcasts defined as a digital, **audio-only**, recording of a radio broadcast or similar program, made available on the internet for downloading to personal audio player or smart device.

**Outcomes:**

- Studies had to explicitly include at least one mental health outcome *and/or* physical health *and/or* health behaviour outcome:

**Mental health outcome:**

- ***Mental illness:*** operationalized by WHO as mental disorder, psychosocial disabilities and other mental states associated with significant distress, impairment to functioning, or risk of self-harm (*refer* *pg. 38 WHO, transferring mental health for all*).
  - ***Mental health:*** “a state of well-being in which the individual realizes his or her own abilities, can cope with the normal stresses of life, can word productively and fruitfully, and is able to make a contribution to his or her community” (WHO, 2001). For example, quality of life, self-esteem, self-concept, self-worth, self-perception, mood, resilience, wellness or happiness, mental fitness, emotional stability, satisfaction, affect, flourishing,

**Physical health outcome:**

- ***Physical health:*** these may include weight status (e.g., weight loss, weight reduction, weight management, weigh control), metabolic risk factors (blood pressure, hyperglycemia [high blood glucose], hyperlipidemia [high levels of fat in the blood]), back problems, arthritis, asthma, diabetes mellitus, heart, stroke, (cardio)vascular disease, osteoporosis, chronic obstructive pulmonary disease, cancer, kidney disease, others to be included through discussion.
  - ***Other health behaviours:*** smoking, alcohol use, recreational drug use, nutrition, and dietary behaviours (e.g., poor diet, nutrition intake), sleep, physical activity (e.g., physical training, exercise, fitness, resistance training, strength training, lifestyle, aerobic) sedentary behaviour (physical inactivity),
  - *Studies that did not include an intervention designed to test physical and/or mental health (e.g., mechanistic-experimental studies with no clear intent to create long-term behaviour change outcomes, those studies focused on educating health care practitioners, or those studies targeting patient education to improve self-efficacy of chronic disease management) were excluded.*

**References:**

<https://www.abs.gov.au/statistics/health/health-conditions-and-risks/national-health-survey-first-results/latest-release#chronic-conditions>

<https://www.racgp.org.au/getattachment/bb78b780-1c37-498a-8ba3-b24a1a4288d9/Smoking-nutrition-alcohol-physical-activity-SNAP.aspx>

World Health Organization. (2001). The World Health Report 2001: Mental health: new understanding, new hope.

World Health Organisation. (2022). World mental health report: transforming mental health for all.
