# Supplementary material for: Podcasts in Mental, Physical, or Combined Health Interventions for Adults: Scoping Review
Source: J Med Internet Res. 2025 May 7;27:e63360. doi: 10.2196/63360 (PMC12096026; doi:10.2196/63360)
Supplement: Multimedia Appendix 3 [file jmir_v27i1e63360_app3.docx]

**Supplementary material (S3):**

**Podcasts in Mental and/or Physical Health Interventions for Adults: A Scoping Review**

**Syntax Search Strategy**

| **Database** | **Search Strategy/ Syntax** | **Limits** |
| --- | --- | --- |
| MEDLINE | (Podcast*).af. and ({intervention*} or {outcome*} or {program*} or {trial*}).mp. | Limit to (English Language and Humans) |
| Psyc Info | (Podcast*).af. and ({intervention*} or {outcome*} or {program*} or {trial*}).mp. | Limit to (English language and Human) |
| EMBASE | (Podcast*).af. and ({intervention*} or {outcome*} or {program*} or {trial*}).mp. | Limit to (English Language and Human) |
| CINAHL | TX (Podcast*) and ({intervention*} or {outcome*} or {program*} or {trial*}) | Limit to (English Language and Human) |
| SCOPUS | All fields: (Podcast*)  and  Article title, abstract, keywords: ({intervention*} or {outcome*} or {program*} or {trial*}) | Limit to (English and Humans) |
| Cochrane Library (CENTRAL) | (Podcast*)  and  ({intervention*} or {outcome*} or {program*} or {trial*}):ti,ab,kw  (Word variations have been searched) | No limits set |

### Appendix II: Data extraction instrument

**Data Extraction Template**

| **Scoping Review Details** | |
| --- | --- |
| **Evidence Source Details and Characteristics** | |
| Citation details |  |
| *Author/s* |  |
| *Year of Publication* |  |
| *Title* |  |
| Country |  |
| Context (e.g., setting) |  |
| **Participants** | |
| *Sample size* |  |
| *Gender*   - *% female* |  |
| *Age:*   - *range* - *mean* |  |
| *Exclusion criteria* |  |
| **Intervention details** | |
| Study Design   - *Type* - *Comparison group Y/N* |  |
| Focus of intervention   - *Mental health* - *Physical health* - *Both* |  |
| Duration |  |
| Targeted outcome measures (list all assessed) |  |
| End-user engagement in design |  |
| Theoretical framework described |  |
| **Podcast characteristics** | |
| Podcast:   - *Podcast only* - *Podcast+other components* |  |
| Number of podcasts |  |
| Duration of each podcast episode |  |
| Frequency of podcasts |  |
| Podcast format (e.g., single presenter, presenter & producer, panel) |  |
| Podcast Content:   - *Native (new) content creation* - *Existing podcast* |  |
| Additional comments on podcast acceptability |  |
