# Supplementary material for: Podcasts in Mental, Physical, or Combined Health Interventions for Adults: Scoping Review
Source: J Med Internet Res. 2025 May 7;27:e63360. doi: 10.2196/63360 (PMC12096026; doi:10.2196/63360)
Supplement: Multimedia Appendix 4 [file jmir_v27i1e63360_app4.docx]

# **Supplementary material (S4): Extraction template**

**Podcasts in Mental and/or Physical Health Interventions for Adults: A Scoping Review**

# **Evidence Source Details and Characteristics**

## **Citation details**

**Lead author and contact details:**

| **Lead author (name):** |  |
| --- | --- |
| **Contact details (listed on article):** |  |
| **Other (e.g., a subsequent author is contact person):** |  |

**Title of study:**

Title of paper / abstract / report that data are extracted from

**Country in which the study conducted:**

**Target population (e.g., women during pregnancy, men with depression):**

## **Participants**

**Sample size (N= ;based on numbers reported in demographics /baseline table):**

**Gender (% female):**

**Age (report to 1 decimal place)**

|  | **Range** | **Mean** | **SD** |
| --- | --- | --- | --- |
| **Age** |  |  |  |

**Inclusion criteria (list):**

|  |
| --- |

**Exclusion criteria (list):**

|  |
| --- |

## **Intervention details**

**Type of article:**

| 1. Published paper 2. Grey literature: Thesis 3. Grey literature: Registered clinical trial 4. Grey literature: Conference abstract 5. Unclear 6. Other |
| --- |

**Study design:**

| 1. Pre-test – Post-test (measures on an outcome are taken before and after the intervention 2. Quasi-experimental (QE) / pseudo-randomised trial (PRT): QE: participants allocated to an intervention or control group, using a non-random method (e.g., clinician preference / availability). PRT: allocated to an intervention group or control group, using a pseudo-random method (e.g., alternate allocation, allocation by days of the week or odd-even numbers). 3. Randomised (controlled) trial (RCT) (participants allocated to either an intervention group or a control group, using a random mechanism) 4. Unclear 5. Not reported 6. Other |
| --- |

**Summary of study arms (brief description of each):**

|  |
| --- |

**Primary outcome (measure) (NB: leave blank if NO primary outcome is clearly specified):**

|  |
| --- |

**Intervention duration:**

|  |
| --- |

**List assessment periods (relative to baseline) (e.g., baseline, 3-months post-baseline, 6-months post-baseline):**

|  |
| --- |

## **Podcast characteristics**

**Podcast arm 1**

**Name of group (e.g., described in methods section of article):**

**Podcast:**

| 1. Podcast only 2. Podcast + other (multi component) |
| --- |

**Theoretical framework described (Podcast arm 1):**

|  |
| --- |

**End-user engagement in design (Podcast arm 1) No / Yes (if yes, describe involvement):**

|  |
| --- |

**Number of podcasts included (Podcast arm 1):**

|  |
| --- |

**Average duration of each podcast episode (Podcast arm 1):**

|  |
| --- |

**Frequency of podcasts (Podcast arm 1) (e.g., once or twice per week):**

|  |
| --- |

**Podcast format (Podcast arm 1):**

| 1. Single presenter 2. Two people (e.g., Presenter & Producer. If known, describe in "other") 3. Three or more people (e.g., Presenter & Panel. If known, describe in "other") 4. Unclear (podcasts description general by nature, but not explicitly stated) 5. Not reported 6. Other |
| --- |

**Content of podcasts (Podcast arm 1) (describe relevant details in "other" e.g., website or name of podcast):**

| 1. Native content creation (developed by the research team) 2. Existing podcast (developed by people outside of the research team) 3. Unclear (podcasts description general by nature, but not explicitly stated) 4. Not reported 5. Other |
| --- |

**Topics covered in podcasts (Podcast arm 1) (list using numbers):**

**Podcast arm 2 (if applicable)**

**Name of group (e.g., described in methods section of article):**

**Podcast:**

| 1. Podcast only 2. Podcast + other (multi component) |
| --- |

**Theoretical framework described (Podcast arm 2):**

|  |
| --- |

**End-user engagement in design (Podcast arm 2) No / Yes (if yes, describe involvement):**

|  |
| --- |

**Number of podcasts included (Podcast arm 2):**

|  |
| --- |

**Average duration of each podcast episode (Podcast arm 2):**

|  |
| --- |

**Frequency of podcasts (Podcast arm 2) (e.g., once or twice per week):**

|  |
| --- |

**Podcast format (Podcast arm 2):**

| 1. Single presenter 2. Two people (e.g., Presenter & Producer. If known, describe in "other") 3. Three or more people (e.g., Presenter & Panel. If known, describe in "other") 4. Unclear (podcasts description general by nature, but not explicitly stated) 5. Not reported 6. Other |
| --- |

**Content of podcasts (Podcast arm 2) (describe relevant details in "other" e.g., website or name of podcast):**

| 1. Native content creation (developed by the research team) 2. Existing podcast (developed by people outside of the research team) 3. Unclear (podcasts description general by nature, but not explicitly stated) 4. Not reported 5. Other |
| --- |

**Topics covered in podcasts (Podcast arm 2) (list using numbers):**

|  |
| --- |

**Process evaluation data of podcast engagement, including:**

**Barriers to use and implementation (e.g., "a circumstance or obstacle that keeps people or things apart or prevents communication or progress" [Lexico, n.d.] in relation to podcast/s engagement)?**

|  |
| --- |

**Facilitators to use and implementation (e.g., "a person or thing that makes something possible" [Lexico, n.d.] something that aids, helps, enhances engage with podcast/s)?**

|  |
| --- |

**Usage data (quantitative if reported, or qualitative if not specifically reported but mentioned in article)?**

|  |
| --- |

**Other podcast process evaluation data (not already captured):**

|  |
| --- |
