# Supplementary material for: Podcasts in Mental, Physical, or Combined Health Interventions for Adults: Scoping Review
Source: J Med Internet Res. 2025 May 7;27:e63360. doi: 10.2196/63360 (PMC12096026; doi:10.2196/63360)
Supplement: Multimedia Appendix 5 [file jmir_v27i1e63360_app5.docx]

**Supplementary Table 1: Key characteristics of included evidence sources**

| **First author (year); Country** | **Participant population** | **Sample size, % female, age range, Mean (SD) age** | **Study Design**  **(intervention assessment periods)** | **Study aim and primary outcome (measure/s)** | **Brief description of study arms** | **Podcast component** |
| --- | --- | --- | --- | --- | --- | --- |
| **Peer reviewed studies** | | | | | | |
| Anderson (2017); Australia^b^ | Women | - *n* = 225 - 100% F - 40-65 years - M (SD) age: 50.9 (NR) years | Pseudorandomised controlled trial (baseline; 12-weeks) | Test efficacy of a wellness intervention delivered across different modes in women in midlife going through menopause.  *Primary outcome:*   - Menopausal symptoms (Green Climacteric Scale) - Health-related Quality of Life (Short Form 12-item) | **Intervention 1:** Online independent accessed Women’s Wellness Program website and downloadable resources. | Podcast + other |
|  |  |  |  |  | **Intervention 2:** Face-to-face with health professional support | No podcast |
|  |  |  |  |  | **Intervention 3:** Online with health professional support | Podcast + other |
| Bangia (2014); United States of America | Grocery shoppers | - *n* = 56 - 79% F - M (SD) age: 41.0 (15.3) years | Pre-test -- Post-test (baseline; post- shopping) | Pilot test if a podcast intervention at point-of-purchase increases awareness and purchase of Omega-3 rich foods.  *Primary outcome not specified* | **Intervention:** Omega-3 information podcast | Podcast only |
| Bangia (2017); United States of America | Grocery shoppers | - *n* = 173 - 76% F - 18-80 years - M (SD) age: 50.4 (13.8) years | Interrupted-time series design  (6-months pre-baseline, with data recorded each month, until 6-months post-baseline) | Examine if a podcast intervention at point-of-purchase results in Omega-3 rich foods from 6-months prior to 6-months post intervention.  *Primary outcome:*   - Total food purchases of Omega-3 rich foods | **Intervention:** Omega-3 information podcast | Podcast only |
| Cai (2023); United States of America | Pregnant, nulliparous, low-risk women | - *n* = 153 - 100% F - 18-50 years - M (SD) age: 29.9 (NR) years | RCT (baseline; post- ≤1 week of delivery) | Test a labour education podcast on personal perception of control and patient satisfaction during childbirth.  *Primary outcome:*   - Personal perception of control and patient satisfaction during childbirth (Labour Agentry Scale) | **Intervention:** labour-related podcast education | Podcast only |
|  |  |  |  |  | **Control/Comparator:** usual standard routine prenatal education | No podcast |
| Davies (2021); Australia | Healthy young adults in the university community | - *n* = 93 - 63.4% F - 18-41 years - M (SD) age: 21.8 (NR) years | RCT (baseline; post-intervention) | Test sham mindfulness to establish whether placebo effects contribute to mindfulness-based interventions for pain.  *Primary outcome:*   - Pain intensity and pain unpleasantness (self-report, Numeric Rating Scale) | **Intervention:** Mindfulness | No podcast |
|  |  |  |  |  | **Intervention:** Sham mindfulness | No podcast |
|  |  |  |  |  | **Control/Comparator:** No treatment | Podcast only |
| Davies (2022); Australia^b^ | Healthy young adults in the university community | - *n* = 153 - 72.5% F - 18-60 years - M (SD) age: 22.8 (NR) years | RCT (baseline; post-intervention) | Examine the role of mindfulness and placebo processes underlying mindfulness-based pain attenuation.  *Primary outcome:*   - Pain unpleasantness (self-report, Numeric Rating Scale) | **Intervention:** Mindfulness treatment and told mindfulness | No podcast |
|  |  |  |  |  | **Intervention:** Mindfulness treatment and told sham | No podcast |
|  |  |  |  |  | **Intervention:** Sham treatment and told mindfulness | No podcast |
|  |  |  |  |  | **Intervention:** Sham treatment and told sham | No podcast |
|  |  |  |  |  | **Control/Comparator:** No treatment | Podcast only |
| Dunn (2019); United States of America | Adults with overweight or obesity | - *n* = 43 - 90.7% F - M (SD) age: 42.4 (12.4) years | RCT (baseline; 6-weeks; 6-months) | Test two mobile-enabled dietary monitoring apps on tracking frequency, and weight loss via a remotely delivered behavioural weight-loss intervention.  *Primary outcome:*   - Weight change (kg) - Diet intake (self-report via app) | **Intervention:** diet intake monitoring via photo app (MealLogger) + theory-based podcasts | Podcast + other |
|  |  |  |  |  | **Control/Comparator:** diet intake monitoring via standard app (Calorie Counter by FatSecret) + theory-based podcasts | Podcast + other |
| Edwards (2021) and Shaw (2022); United States of America^a^ | Women (aged 40 to 60 years) | - *n* = 30 - 100% F - 40-60 years - M (SD) age: 48.8 (7.42) years | Qualitative: Semi-structured interviews post-podcast | Examine the impact of podcast on women’s menopause-related knowledge, understanding and communication practices.  *Primary outcome:*   - Semi-structured interviews were analysed thematically | **Intervention**: menopause podcast series | Podcast only |
| Hales, 2016; United States of America | Adults with overweight or obesity | - *n* = 51 - 82.4% F - M (SD) age: 46.2 (12.4) years | RCT (baseline; 12-weeks) | Test efficacy of a researcher-developed mobile app (Social Pounds Off Digitally) behavioural weight-loss intervention, compared to a commercially available tracking app.  *Primary outcome:*   - Weight loss (kg) | **Intervention:** Social Pounds Off Digitally app + theory-based podcasts | Podcast + other |
|  |  |  |  |  | **Control/Comparator:** standard diet monitoring and weight loss app (Calorie Counter by FatSecret) + theory-based podcasts | Podcast + other |
| Huberty (2020); United States of America^b^ | Adult patients with haematological cancer (myeloproliferative neoplasm) | - *n = 80* - 91% F - NR - M (SD) age: 56.1 (10.9) years | Pre-test – post-test feasibility (baseline; 12-weeks) | Test a health education podcast via a smartphone app.  *Primary outcome:*   - Feasibility benchmarks associated with podcast and app usage. | **Intervention**: listen to health education podcasts (~60 min per week for 12 weeks) | Podcast only |
| Kanstrup (2021); Sweden | Trauma-exposed patients in a hospital emergency department | - *n* = 41 - 56% F - 19-76 years - M (SD) age: 46.2 (15.7) years | RCT (baseline; 1-week; 1-month; 5-weeks; 3-months; 6-months) | Test feasibility and effects of a brief behavioural intervention to reduce intrusive memories in trauma-exposed patients.  *Primary outcome:*   - Number of intrusive memories of traumatic event (daily diary, week 1) | **Intervention:** memory reminder cue + visuospatial cognitive “Tetris” task with mental rotation instructions using Tetris app | No podcast |
|  |  |  |  |  | **Control/Comparator:** received podcast ≤72 hours of Emergency Department presentation. | Podcast only |
| Laird (2022); United  States of America^b^ | Adults | - *n* = 58 - 65.5% F - 40-65 years - M (SD) age: 51.5 (NR) years | RCT feasibility (baseline; 4-weeks) | Test feasibility of a consumer-based mediation app to reduce stress and improve stress-related outcomes of middle-aged adults.  *Primary outcome:*   - Feasibility of Calm app:   - acceptability and demand (Bowen framework) | **Intervention:** Calm app-based wellness meditation intervention (≥10 minutes per day) | No podcast |
|  |  |  |  |  | **Control/Comparator:** listen to health education podcasts (≥10 minutes per day) | Podcast only |
| Lui (2021),  Lui (2022) & Wilcox (2022); United States of America^a^ | White and African American pregnant women with overweight and obesity | - *n* = 219 - 100% F - 18 to 44 years - M (SD) age: 29.7 (5) years | RCT (baseline ~≤18 weeks gestation; 32-weeks’ gestation; 6-months post-partum, 12-months post-partum) | Test a behavioural lifestyle intervention for African American and white women living with overweight and obesity from pregnancy to post-partum.  *Primary outcome:*   - Weight retention outcomes:   - Gestational Weight Gain (kg)   - Institute of Medicine weight outcomes (excessive, inadequate, adequate) | **Intervention:** Behavioural lifestyle: in-depth counselling, brief telephone counselling, behavioural podcasts | Podcast + other |
|  |  |  |  |  | **Control/Comparator:** Standard usual prenatal care + six monthly mailings, standard podcasts. | Podcast + other |
| Mailey (2016); United States of America | Working mothers | - *n* = 69 - 100% F - M (SD) age: 35.9 (5.1) years | RCT feasibility (baseline; 8-weeks; 16-weeks) | Test feasibility of online intervention (Fit Minded) for working mothers.  *Primary outcome not specified* | **Intervention:** Enhanced: Fit Minded Working Moms program (online) + additional group dynamics content | Podcast + other |
|  |  |  |  |  | **Control/Comparator:** Standard: Fit Minded Working Moms program (online) | Podcast + other |
| Mailey (2019); United States of America | Military spouses | - *n* = 231 - 100% F - M (SD) age: 32.3 (NR) years | Quasi-experimental / Pseudo-randomised trial (baseline; 10-weeks) | Examine an interactive, theory-based web-delivered intervention, compared to  *Primary outcome not specified* | **Intervention:** online interactive wellbeing program (InDependent but not Alone) | Podcast + other |
|  |  |  |  |  | **Control condition:** standard, educational online intervention (Operation Live Well) | No podcast |
| Militello (2021); United States of America | Perinatal women (pregnant or infant aged ≤1 year). | - *n* = 19 - 100% F - 25-34 years - M (SD) age: NR | Pre-test – post-test feasibility (baseline; 2-weeks) | Test feasibility of using voice technology to support perinatal health and infant care practices in women.  *Primary outcome not specified* | **Intervention**: received Self-Management Intervention-Life Essential voice interactive app (e.g., Siri or Alexa) + daily podcast. | Podcast only |
| Seib (2022); Australia | Women previously treated (≤ 2 years of) for breast, blood or gynaecological cancer | - *n* = 351 - 100% F - M (SD) age: 53 (8.8) years | Multi-site (3x Australian hospitals) RCT (baseline; 12-weeks; 24-weeks) | Test a health promotion intervention on health-related quality of life in women diagnosed with cancers associated with treatment-induced menopause.  *Primary outcome:*   - Health-related quality of life (Functional Assessment of Cancer Therapy-General) | **Intervention:** received iBook, journal, web interface, virtual health consultations | Podcast + other |
|  |  |  |  |  | **Control/ Comparator:** Standard usual | No podcast |
| Shaw (2013); United States of America | Veterans receiving intensive outpatient substance abuse treatment | - *n* = 18 - 20% F - 29-62 years - M (SD) age: 49 (NR) years | Pre-test – post-test (baseline; 2-weeks) | Evaluate feasibility of mobile media players as a resource to help veterans in recovery for alcohol abuse and dependence disorder.  *Primary outcome not specified* | **Intervention:** podcasts (as supplementary materials to standard care) over a two-week period |  |
| Shaygan (2021); Iran^b^ | Adult patients hospitalised with coronavirus disease | *n* = 4843.8% FM (SD) age: 36.7 (11.8) years | Cluster RCT. Different hospital wards formed different clusters. (baseline; 2-weeks) | Evaluate feasibility of an online multimedia psychoeducational intervention and perceived stress.  *Primary outcome:*   - Resilience (Connor-Davidson resilience scale) | **Intervention:** Online multimedia psychoeducational: daily evidenced-based modules via WhatsApp. | Podcast + other |
|  |  |  |  |  | **Control/Comparator:** telephone-base counselling from the psychological team | No podcast |
| Stork (2019); Canada | Insufficiently active adults, inexperienced in Sprint Interval Training | - *n* = 24 - 50% F - M (SD) age: 24.1 (NR) years | RCT  Randomised, cross-over design (5 lab sessions across 2-3 weeks) | Test psychological, psychophysical, and physiological effects of motivational music during a low-volume sprint interval training exercise.  *Primary outcome not specified* | **Intervention:** 3 x 20 seconds “all-out” sprints listening to motivational music | No podcast |
|  |  |  |  |  | **Control/ Comparator:** 3 x 20 seconds “all-out” sprints listening to a podcast | Podcast only |
|  |  |  |  |  | **Control/Comparator:** 3 x 20 seconds “all-out” sprints listening to no-audio | No podcast |
| Tavakolizadeh (2021); Iran^b^ | Patients with coronary heart diseases | - *n* = 40 - 25% F - M (SD) age: 51.6 (NR) years | RCT (baseline; 8-weeks; 12-weeks) | Test the effects of a bioenergy economy-based psycho-education package on improvement of vegetative function, forgiveness, and quality of life.  *Primary outcome:*   - Vegetative functions (vegetative function checklist) - Forgiveness (Forgiveness Likelihood Scale) - Quality of Life (World Health Organization Quality of Life – BREF) | **Intervention:** routine cardiovascular medications and routine care + trained using an audio Bio Energy Economy-based psychoeducation package | Podcast + other |
|  |  |  |  |  | **Control/Comparator:** routine cardiovascular medications and care | No podcast |
| Turner McGrievy (2009); United States of America | Adults with overweight or obesity | - *n* = 78 - 73.1% F - M (SD) age: 38.6 (NR) years | RCT  Baseline; 12-weeks post-baseline | Test a weight-loss researcher-developed (theory-based) podcast intervention, compared to a standard currently available podcast (not theory-based).  *Primary outcome:*   - Weight (kg) | **Intervention:** Enhanced podcast: receive a theory-based podcast on weight loss | Podcast + other |
|  |  |  |  |  | **Control/Comparator:** receive a currently available weight loss podcast | Podcast + other |
| Turner McGrievy (2011); United States of America | Adults with overweight or obesity | - *n* = 96 - 75% F - M (SD) age: 42.9 (NR) years | RCT  Baseline; 3-months post-baseline; 6-months post-baseline | Examine whether a combination of podcasting, mobile support communication and mobile diet monitoring can assist people in weight loss.  Primary outcome:   - Change in body weight (%) | **Intervention:** Theory-based podcast + self-monitor dietary intake and physical activity app (Calorie Counter by Fat Secret) + communications via social media (Twitter) | Podcast + other |
|  |  |  |  |  | **Control/Comparator:** Theory-based podcast + book with calorie and fat gram amounts of food to self-monitor dietary intake. | Podcast + other |
| Turner McGrievy (2017); United  States of America | Adults with overweight and obesity | - *n* = 12 - 92% F - M (SD) age: 54.1 (NR) years | Pre-test -- Post-test  Baseline; 4-weeks post-baseline | Evaluate feasibility and usability of mobile Bite Counter, and behavioural challenges on diet and physical activity outcomes.  *Primary outcome:*   - Weight (kg) | **Intervention**: self-monitor number of bites per day using the Bite Counter, attend weekly group sessions, and listen to podcasts. | Podcast + other |
| Turner McGrievy (2017); United States of America | Adults with overweight or obesity | - *n* = 81 - 82.7% F - M (SD) age: 48.1 (NR) years | RCT  Baseline; 3-months post-baseline; 6-months post-baseline | Examine the use of two different mobile dietary self-monitoring methods for weight loss.  *Primary outcome:*   - Weight loss (kg) | **Intervention:** Theory-based podcasts + self-monitor daily energy intake via standard app (Calorie Counter by FatSecret) | Podcast + other |
|  |  |  |  |  | **Control/Comparator:** Bite: TBP + turn on and wear Bite Counter device whilst eating | Podcast + other |
| Wahbeh (2016); United States of America | Cognition and mood in older adults | - *n* = 16 - 50% F - 65-90 years - M (SD) age: 76.2 (7.4) years | RCT  Baseline; 6-weeks post-baseline | Evaluate feasibility and acceptability of an internet mindfulness mediation intervention and an internet health and wellness education program.  *Primary outcome:*   - Feasibility and acceptability (Client Satisfaction Questionnaire) | **Intervention:** Internet Mindfulness Meditation: standardised and structured evidenced-based program | No podcast |
|  |  |  |  |  | **Control/Comparator:** Internet Education: general health video, materials + home practice podcasts | Podcast + other |
| **Grey Literature – PhD Dissertations / Theses** | | | | | | |
| Dahl (2013) [30]; United States of America | Pregnant women | - *n* = 87 - 100% F - 20 to 35 years - M (SD) age: 30.1 (NR) years | RCT  Baseline (≤20 weeks gestation); ~32-34 weeks gestation (post-intervention) | Examine effects of a mobile health intervention, Healthy Motivations for Moms-To-Be, targeting several health behaviours and gestational weight gain during pregnancy and post-partum.  *Primary outcome:*   - Total gestational weight gain (self-report + photo of bathroom scales display) | **Intervention:** educational materials + personalised Healthy Eating and Physical Activity mobile app + complete behavioural challenges on 5 of 7 days per week | Podcast + other |
|  |  |  |  |  | **Control/Comparator:** educational materials + personalised Stress Reduction And Management mobile app + complete behavioural challenges on 5 of 7 days per week | Podcast + other |
| Duffy (2013); United States of America | Primary caregiver / parents of children (aged 2-6 years) with diagnosed epilepsy (≥ 6-months since diagnosis) | - *n* = 46 - 80.4% F | RCT (baseline; post-discharge from hospital at 1-week; 4-6 weeks; 10-12 weeks) | Test the efficacy of Creating Opportunities for Parent Engagement intervention for parents of children with epilepsy and other neurological conditions.  *Primary outcome:*   - Parental Belief (Parental Beliefs Scale) - Depression (Beck Depression Inventory – II) - Anxiety (State-Trait Anxiety Inventory) | **Intervention:** Creating Opportunities for Parent Engagement three-phase care:  Phase I: information delivered in writing & via audiotape, MP3 download or audiotape.  Phase II: telephone call 3-days post-discharge  Phase III: information delivered in writing & via audiotape, MP3 download or audiotape | Podcast + other |
|  |  |  |  |  | **Control/ Comparator:** care as usual. Received information, follow-up telephone call, book and questionnaires. | No podcast |
| Dunston (2020): United States of America | University graduate nursing students | - *n* = 30 | Pre-test – Post-test (baseline; 12-weeks) | Examine the use of the *Sanvello* app on depression, anxiety and stress in graduate nurses.  *Primary outcome:*   - Anxiety symptoms (Generalised Anxiety Disorder scale – 7) - Depressive symptoms (Personal Health Questionnaire – 8) - Perceived stress (Perceived Stress Scale) | **Intervention:** all received access to Sanvello application across semester. | Podcast only |
| Kazan (2018); Australia | Adults who had separated from a romantic relationship in the last 6-months | - *n* = 124 - 90% F - 18-60 years - M (SD) age: 31.2 (NR) years | RCT (baseline; 3-weeks) | Test ‘*MindCast’*, a web-based, audio podcast for adults who had recently separated from an intimate partner relationship.  *Primary outcome:*   - Suicidal ideation (Suicidal Ideation Attributes Scale) - Depression (Patient Health Questionnaire 9-item) | **Intervention:** *‘MindCast’*: Participants in the intervention condition were given three weeks to complete the podcast program. | Podcast only |
|  |  |  |  |  | **Control/Comparator:** Waitlist |  |
| Nkwocha (2022); United States of America | Adults with overweight or obesity | - *n* = 15 - 60% F - 26-65 years - M (SD) age: 54.0 (10.7) years | Quasi-experimental pre-test – post-test (baseline; 4-weeks) | Evaluate the effects of using the digital health monitoring intervention combined with a podcast for weight management and maintenance.  *Primary outcome:*   - Difference in mean weight between baseline to post-implementation | **Intervention:** Theory-based podcasts + Calorie Counter FatSecret app | Podcast + other |
|  |  |  |  |  | **Control/ Comparator:** Care as usual (current practice) | No podcast |
| **Grey Literature – Published Protocols** | | | | | | |
| Huberty (2022); United States of America | Patients with chronic haematological cancer | - *n* = 276 | Protocol for RCT (baseline; 8-weeks; 20-weeks) | Test the efficacy of daily mediation delivered via Calm app compared with a health education podcast control group in improving sleep disturbance.  *Primary outcome:*   - Change in sleep disturbance (Insomnia Severity Index) | **Intervention:** App-based wellness: app-based meditation intervention (≥10 minutes per day) delivered via Calm (pre-existing app) | No podcast |
|  |  |  |  |  | **Control/Comparator:** Health education podcast app: listen to podcasts (≥10 minutes per day) | Podcast only |
| Turner-McGrievy (2020); United States of America | Adults who are African American with overweight or obesity | - *n* = 130 - 18 to 65 years | Protocol for RCT (baseline; 6-months; 12-months; 24-months) | Nutritious Eating with Soul examines the impact of two different culturally tailored diets on changes in risk factors for cardiovascular disease.  *Primary outcome:*   - Change in bodyweight and cardiovascular risk factors (lipids, blood pressure, glucose and insulin) at 12-months | **Intervention:** follow a whole-foods, plant-based vegan diet + weekly classes from 0-6 months; bi-weekly classes from 6-12 months; monthly meetings for 12-24 months. Facebook groups, podcasts & newsletters from 6-months. | Podcast + other |
|  |  |  |  |  | **Intervention:** follow a low-fat omnivorous diet + weekly classes from 0-6 months; bi-weekly classes from 6-12 months; monthly meetings for 12-24 months. Facebook groups, podcasts & newsletters from 6-months. | Podcast + other |
| Woodworth (2023); United States of America | Adult caregivers of people with Alzheimer disease and related dementias | - *n* = 80 | Protocol Feasibility RCT (baseline;12-weeks; 20-weeks) | Evaluate feasibility of two free mobile apps.  *Primary outcome:*   - Caregiver stress (measure not specified) | **Intervention:** Healthy Minds Program is a self-guided meditation app that includes training in mindfulness. | No podcast |
|  |  |  |  |  | **Control/ Comparator:** Access Wellness app self-guided caregiver-related, knowledge-based podcasts. | Podcast only |
| **Grey Literature – Registered Clinical Trial** | | | | | | |
| Arnold (n.d.); Australia | Healthy adults | - *n* = 60 - 60% F - 19-63 years - M (SD) age: 41.4 (NR) years | RCT – Clinical Trial identifier: ACTRN12622001064796  (baseline; 6-weeks; 12-weeks; 6-months) | Test an online running intervention on motivational and psychological factors related to running maintenance.  *Primary outcome:*   - Feasibility (benchmarks unclear) | **Intervention:** Online – weekly content targets aspects of motivation, psychological needs, and self-efficacy to promote running. | Podcast + other |
|  |  |  |  |  | **Control/Comparator:** Condensed – basic content delivered in the first 2-weeks + track running activity on Strava. | No podcast |
| Fitzpatrick (n.d.); Australia | Adult family members/ friends supporting a current, or previously employed, paramedic experiencing mental ill-health or suicidal distress | - Not reported | RCT – Clinical Trial identifier: ACTRN12622001547730  (baseline: 8-weeks; 6-months) | Evaluate feasibility and acceptability of an online intervention for family and friends supporting a paramedic experiencing mental ill-health or suicidal distress.  *Primary outcome:*   - Change in caregiver:   - Burden (Burden Scale for family caregivers)   - Coping self-efficacy (Coping Self-efficacy Scale)   - Perception of social support (Perceived Social Support Questionnaire) | **Intervention:** *Minds Together*: complete an Online Support Program (4 core sections + 6 optional in-depth topics) such as, suicidal distress, mental health, coping skills and caregiving support. | Podcast + other |
|  |  |  |  |  | **Control/Comparator:** Waitlist: will receive access to the program in 8 weeks. | No podcast |
| Hammer (n.d.); United States of America | Healthy adults in Active-Duty Service Members Serving (Striker Brigade Combat Teams, Positions of platoon leader or below) | - *n = 2297* | RCT – Clinical Trial identifier: NCT04152824 (baseline; 6-months) | Test an evidence-based leadership support intervention, Resilience-Supportive Leadership Training.  *Primary outcome:*   - Leader reactions to Resilience-Supportive Leadership Training (measure not specified) | **Intervention:** Leader Intervention  Leaders in the intervention group will go through the Resilience-Supportive Leadership Training | Podcast + other |
|  |  |  |  |  | **Control/Comparator:** Leaders in the control group will be usual practice | No podcast |
| Jácome-Hortua (n.d.); Columbia | Adults attending cardiac rehabilitation sessions | - Not reported | RCT – Clinical Trial identifier: ACTRN12622001446752  (baseline; 4-week; 3-months; 6-months; 12-months) | Examine the effects of an educational intervention delivered via WhatsApp, compared to standard (usual) care.  *Primary outcome:*   - Cardiovascular risk factor knowledge score (Knowledge questionnaire designed and validated by research team) | **Intervention:** WhatsApp: Additional education information sent daily via WhatsApp (e.g., videos, images and/or podcasts) with 4 participants in each WhatsApp group. | Podcast + other |
|  |  |  |  |  | **Control/Comparator:** Usual care (supervised exercise sessions and short educational lectures) | No podcast |
| Janevic (n.d.); United States of America | Adults with elevated physical or psychosocial symptoms | - Target *n* = 456 - ≥50 years | RCT – Clinical Trial identifier: NCT05451589  (baseline; 2-months; 8-months) | Test if *‘Re-Engaging in Self-care, Enjoying Today’* multi-component intervention will improve psychosocial and physical functioning.  *Primary outcome:*   - Change in Mental (Psychosocial Summary Score) | **Intervention:** *‘Re-Engaging in Self-care, Enjoying Today’* telephone-based program + podcast series + activity tracking + individual & group sessions | Podcast + other |
|  |  |  |  |  | **Control/Comparator:** Wellness check: educational information and individual wellness check phone calls to screen for unmet social needs |  |
| Klein (n.d.); United States of America | Young adults who vape nicotine | - 18 to 24 years | RCT – Clinical Trial identifier: NCT04974580 (baseline; 3-months) | Test intervention components to help young adults quit vaping. All participants will receive 2 counselling calls. Plus, one intervention condition.  *Primary outcome:*   - 7-day point prevalence vaping abstinence (Self-report) | **Intervention:**  Phone coaching only | No podcast |
|  |  |  |  |  | - Digital: phone coaching + digital coaching | Podcast + other |
|  |  |  |  |  | - Phone coaching + Nicotine Replacement therapy | No podcast |
|  |  |  |  |  | - Digital content + Nicotine Replacement therapy | Podcast + other |
| Mikcocka-Walus (n.d.); Australia | Adults with a diagnosis of endometriosis | - Not reported | RCT – Clinical Trial identifier: ACTRN12623000598684p (baseline; 8-weeks post-treatment; 6-months) | Evaluate a modular telehealth-based supportive care program, and its impact on pain and quality of life.  *Primary outcome:*   - Endometriosis-related quality of life (Endometriosis Health Profile 30) - Quality of life (Euroqol 5D-5L) | **Intervention:** ‘CoDeEndo’: complete 8 evidenced-based, self-directed modules on pain, mental health and quality of life in people with endometriosis. | Podcast + other |
|  |  |  |  |  | **Control/Comparator:** Waitlist: usual medical care and offered CoDeEndo at the end of study. | No podcast |
| Phillips (n.d.); United States of America | Adult female breast cancer survivor | - Target *n* = 304 | RCT – Clinical Trial identifier: NCT05931874  (baseline; 24-weeks; 48-weeks) | Test which intervention components increase moderate-to-vigorous physical activity adoption and maintenance. Participants receive core program (Fitbit + Opt2Move App), plus one intervention condition.  *Primary outcome:*   - Physical activity (ActiGraph) | **Intervention:**   - Buddy (train with partner + podcasts every 3-weeks) | Podcast + other |
|  |  |  |  |  | - E-coaching (text messages from coach) | No podcast |
|  |  |  |  |  | - General guided audio mindfulness training | No podcast |
|  |  |  |  |  | - MVPA-specific guided audio mindfulness training | No podcast |
| Phillips (n.d.); United States of America | Young adult cancer survivors | - Target *n* = 304 - 18-39 years | RCT – Clinical Trial identifier: NCT05375162  (baseline; 12-weeks; 24-weeks) | Test which intervention components increase moderate-to-vigorous physical activity adoption and maintenance. Participants receive core program (Fitbit + Opt2Move App), plus one intervention condition.  *Primary outcome:*   - Physical activity (ActiGraph) | **Intervention:**   - Buddy (train with partner + podcasts every 3-weeks) | Podcast + other |
|  |  |  |  |  | - E-coaching (text messages from coach) | No podcast |
|  |  |  |  |  | - General guided audio mindfulness training | No podcast |
|  |  |  |  |  | - MVPA-specific guided audio mindfulness training | No podcast |
| Pirzadeh (n.d.); Iran | Adults with diagnosis of non-alcoholic fatty liver disease by physician | - Not reported - 20-50 years | RCT – Clinical Trial identifier: IRCT20210612051546N1  (baseline; 2-months) | Examine the effects of an education-based intervention on physical activity and diet intake.  Primary outcome:   - Dietary intake (3-day food record) - Physical activity (International Physical Activity Questionnaire) | **Intervention:** education on fatty liver disease, physical activity & healthy eating sent via WhatsApp for 2 months + self-care book & weekly self-assessment tables & photo. | Podcast + other |
|  |  |  |  |  | **Control/Comparator:** No treatment. Follow routine life. | No podcast |
| Pirzadeh (n.d.); Iran | Women who have entered menopausal period | - Not reported - 40-50 years | RCT – Clinical Trial identifier: IRCT20210602051478N1  (baseline; 2-months) | Examine the effect of physical activity on quality of life during menopause.  *Primary outcome:*   - Physical activity (International Physical Activity Questionnaire) - Quality of life (Menopause-Specific Quality of Life) | **Intervention:** fortnightly teaching concepts are sent electronically for participants to review and give feedback + Exercise teaching online x 3 sessions per week. | Podcast + other |
|  |  |  |  |  | **Control/ Comparator:** No treatment | No podcast |
| Amirzadegan (n.d.); Iran | Older adults with ability to use a mobile phone, read, write, see and hear | - Not reported - 60-74 years | RCT – Clinical Trial identifier: IRCT20210427051101N1  (baseline; 8-weeks) | Examine effects of virtual self-care education on coronavirus disease anxiety, among community dwelling elderly.  *Primary outcome:*   - COVID-19 (coronavirus disease) Anxiety (Coronavirus Pandemic Anxiety Scale) | **Intervention:** 6-consecutive tutorials on physical, psychological and social self-care training related to coronavirus disease with follow-up video calls via WhatsApp once every three weeks. | Podcast + other |
|  |  |  |  |  | **Control/Comparator:** No treatment. Once every three weeks a welfare follow-up video call via WhatsApp is conducted. | No podcast |
| Rio (n.d.); Portugal | Adults with ST-segment elevation acute myocardial infarction undergoing primary angioplasty | - Target *n* = 86 - 30-90 years | RCT – Clinical Trial identifier: NCT05244707  (baseline; 4-weeks; 8-weeks; 12-weeks) | Test an intensive program will improve cardiovascular risk predictor parameters used in the Secondary Manifestations of Arterial Disease Risk Score tool.  *Primary outcome:*   - Change in Secondary Manifestations of Arterial Disease risk score | **Intervention:** follow an intensive food and nutrition program of Mediterranean style diet, including individual, face-to-face consultations, telephone contact, text messages, podcasts, videos, nutrition workshops. | Podcast + other |
|  |  |  |  |  | **Control/Comparator:** standard education and care recommended by Nutritional Support Protocol of the Cardiac Rehabilitation Program | No podcast |
| Turner-McGrievy (n.d.); United States of America | Adults with overweight/obesity and ≥3 Type 2 diabetes mellitus risk factors | - Not reported - 18-65 years | RCT – Clinical Trial identifier: NCT05176847  (baseline; 12-months) | Examine long-term sustained weight loss digital intervention in a diverse cohort of adults.  *Primary outcome:*   - Change in Kcals consumed per day (dietary recall); Change in minutes a week of moderate to vigorous physical activity (Moderate-to-Vigorous Physical Activity; accelerometer) | **Intervention:** gamified Mobile Lifestyle Intervention for Food and Exercise app + theory-based podcasts, daily tips, weight & physical activity tracking + gaming social support features in app | Podcast + other |
|  |  |  |  |  | **Control/Comparator:** gamified Mobile Lifestyle Intervention for Food and Exercise app + theory-based podcasts, daily tips, weight & physical activity tracking + non-gaming social support features in app | Podcast + other |
| Weisel (n.d.); Germany | - Not reported | - Not reported | RCT – Clinical Trial identifier: DRKS00027998  (baseline; 1-week; 2-week; 3-weeks; 4-weeks; 8-weeks) | Evaluate the feasibility, acceptability and efficacy of a podcast intervention (Flexplore-ACT more).  *Primary outcome:*   - Change in Psychological Flexibility (Acceptance and Action Questionnaire-II) | **Intervention:** Weekly podcasts | Podcast only |
|  |  |  |  |  | **Control/Comparator:** Waitlist: intervention materials given after an 8-week delay. | No podcast |
| **Grey Literature Conference Abstracts** | | | | | | |
| Peaceman (2017); United States of America | Pregnant women (gestational age ≤ 16 weeks) with overweight or obesity | - *n* = 281 | RCT | Test a diet and lifestyle behavioural intervention designed to adhere to the Institute of Medicine gestational weight gain guidelines positively impacts pregnancy outcomes.   - Gestational Weight Gain | **Intervention:** individualized calorie-specific Dietary Approaches to Stop Hypertension-type diet, physical activity, internet-based self-monitoring of diet adherence, weekly coaching calls, group visits, webinars, and podcasts. | Podcast + other |
|  |  |  |  |  | **Control/ Comparator:** Usual care | No podcast |
| Weisbrod (2019); United States of America | People with celiac disease | - Not reported | Not reported | To develop an educational digital app tool to improve knowledge about gluten-free diet.   - User/s access of app (average number of plugins accessed per app open) | **Intervention**: feasibility of Gluten-Free Diet Digital Resource Centre app with 6 educational plugins (e.g., resources materials) | Podcast + other |

**Key:**

^a^ Information for this study was collated from multiple papers relating to the same study.

^b^ The evidence source was identified through the initial search as a registered clinical trial and has since been published as a peer-reviewed paper.

App = Application

RCT = Randomised controlled trial
