# Supplementary material for: Podcasts in Mental, Physical, or Combined Health Interventions for Adults: Scoping Review
Source: J Med Internet Res. 2025 May 7;27:e63360. doi: 10.2196/63360 (PMC12096026; doi:10.2196/63360)
Supplement: Multimedia Appendix 6 [file jmir_v27i1e63360_app6.docx]

**Supplementary Table 2: Podcast characteristics**

| **Author (year)** | **Study arms using podcasts** | **Podcast source and presenter/s** | **Theoretical framework and end-user engagement in design** | **Number, frequency, average duration of episodes** | **Topics** |
| --- | --- | --- | --- | --- | --- |
| **Peer reviewed studies** | | | | | |
| Anderson (2017) | - Online independent condition - Online supported condition | Source: Pre-existing podcast  Presenters: Not reported | *Theory:* Social Cognitive Theory (Bandura, 1986)  *Engagement:* Not reported | *Number:* Not reported  *Frequency:* Not reported  *Duration:* Not reported | Not reported |
| Bangia (2014) | - Omega-3 information podcast | Source: Researcher developed  Presenters: Not reported | *Theory:* Theory of Reasoned Action  *Engagement:* Not reported | *Number:* 1  *Frequency:* All covered at once  *Duration:* 5 minutes | Health benefits of eating food rich in Omega-3 and strategies for increasing consumption. |
| Bangia (2017) | - Omega-3 information podcast | Source: Researcher developed podcast  Presenters: Not reported | *Theory:* Theory of Reasoned Action  *Engagement:* Feedback from pilot participants | *Number:* 10  *Frequency:* All covered at once  *Duration:* 2:07 minutes | Health benefits of eating food rich in Omega-3 and strategies for increasing consumption. |
| Cai (2023) | - Podcast education condition | Source: Researcher developed podcast  Presenters: Two presenters | *Theory:* Not reported  *Engagement:* Focus groups of recent postpartum patients | *Number:* 7  *Frequency:* All released at once  *Duration:* ~10 to 20 minutes | Childbirth, labour experiences and postpartum recovery. |
| Davies (2021) | - Control condition | Source: Pre-existing podcast  Presenter: Not reported | *Theory:* Not reported  *Engagement:* Not reported | *Number:* 2  *Frequency:* Session 1, Session 6 *Duration:* 20 minutes | Radio content regarding the history and practice of mindfulness |
| Davies (2022) | - Control condition | Source: Pre-existing podcast  Presenter: Not reported | *Theory:* Not reported  *Engagement:* Not reported | *Number:* 2  *Frequency:* Session 1, Session 6 *Duration:* 20 minutes | Radio content regarding the history and practice of mindfulness |
| Dunn (2019) | - Diet monitoring via photo app (MealLogger) condition - Diet calorie counter app (Fat Secret) condition | Source: Researcher developed podcast  Presenter: Not reported | *Theory:* Social Cognitive Theory (Bandura, 1986) and Diabetes Prevention Program  *Engagement:* Not reported | *Number:* 48  *Frequency:* Twice weekly  *Duration:* Not reported | Podcasts included theory-based behavioural weight-loss techniques. |
| Edwards (2021) & Shaw (2022)^a^ | - Menopause information podcast | *Source:* Pre-existing podcast  *Presenters:* Not reported | *Theory:* Storytelling  *Engagement:* lived experience and listener activation | *Number:* 6  *Frequency:* All released at once  *Duration:* 20 minutes | Physiological and psychological impacts of menopause and connectedness with others. |
| Hales (2016) | - Social Pounds Off Digitally app condition - Standard weight loss app control condition | *Source:* Researcher developed podcast  *Presenters:* Not reported | *Theory:* Social Cognitive Theory (Bandura, 1986)  *Engagement:* Not reported | *Number:* 24  *Frequency:* Twice weekly  *Duration*: Not reported | Behavioural strategies to improve nutrition and physical activity with a focus on achieving a healthy weight |
| Huberty (2020) | - Health education Podcast app control condition | *Source:* Pre-existing podcast  *Presenters:* Not reported | *Theory:* Not reported  *Engagement:* Not reported | *Number:* Not reported  *Frequency:* Not reported  *Duration:* Not reported | Nutrition, physical activity, time and stress management, general wellness and life-related topics |
| Kanstrup (2021) | - Control condition | *Source:* Pre-existing podcast  *Presenters:* Not reported | *Theory:* Not reported  *Engagement:* Not reported | *Number:* 1  *Frequency:* Once off  *Duration: ~*10 to 20 minutes | Radio content related to Swedish Philosophy |
| Laird (2022) | - Health Education Podcast App control condition | *Source:* Pre-existing podcast  *Presenters:* Not reported | *Theory:* Not reported  *Engagement:* Not reported | *Number:* Not reported  *Frequency:* Not reported  *Duration:* 10 minutes | Health and wellbeing, but excludes mindfulness, stress, sleep content. |
| Lui (2021), Lui (2022) & Wilcox (2022)^a^ | - Behavioural lifestyle intervention condition | *Source:* Researcher developed podcast  *Presenters:* Two presenters | *Theory:* Social Cognitive Theory (Bandura, 1986), Diabetes Prevention Program  *Engagement:* Not reported | *Number:* 26 (10 pregnancy, 16 postpartum)  *Frequency:* Weekly  *Duration:*   - Pregnancy: ~21 minutes - Postpartum: ~20 minutes | Pregnancy podcasts: diet and weight-related information in pregnancy, and physical activity.  Postpartum podcasts: followed 16 core Diabetes Prevention Program sessions. |
|  | - Control/Comparator: Standard Care condition | *Source:* Pre-existing podcast  *Presenters:* Not reported | *Theory:* Not reported  *Engagement:* Not reported | *Number:* 26 (10 pregnancy, 16 postpartum)  *Frequency:* Weekly  *Duration:*   - Pregnancy: ~28 minutes - Postpartum: ~22 minutes | Healthy pregnancy; fetal and infant development; parenting.  * Neither mailings nor podcasts discussed weight, physical activity, or diet. |
| Mailey (2016) | - Wellbeing podcast | *Source:* Pre-existing podcast  *Presenters:* Not reported | *Theory:* Self Determination Theory (Ryan & Deci, 2000)  *Engagement:* Not reported | *Number:* 8  *Frequency:* Weekly  *Duration:* 20 to 30 minutes | Wellbeing topics including Self-renewal; Stress reduction; Work-life balance; Mindfulness |
| Mailey (2019) | - Online interactive wellbeing program (InDependent But Not Alone) condition | *Source:* Researcher developed podcast  *Presenters:* Not reported | *Theory:* Self Determination Theory (Ryan & Deci, 2000); Behaviour Change Technique Taxonomy (Michie et al., 2013); *Engagement:* Focus groups with military spouses. | *Number:* 3-5 per week  *Frequency:* Weekly  *Duration:* ~10 to 15 minutes | Topics specifically tailored to military spouses: physical activity; nutrition and diet; personal growth or emotional wellbeing. |
| Militello (2021) | - Perinatal health podcast | *Source:* Pre-existing podcast  *Presenters:* Not reported | *Theory:* Cognitive Load Theory; Technology Acceptance Model  *Engagement:* Not reported | *Number:* 26  *Frequency:* Not reported  *Duration:* 3 to 8 minutes | Topics related to sleep, pregnancy, parenting and breastfeeding |
| Seib (2022) | - Intervention condition | *Source:* Not reported  *Presenters:* Not reported | *Theory:* Not reported  *Engagement:* Not reported | *Number:* Not reported  *Frequency:* Not reported  *Duration:* Not reported | Not reported |
| Shaw (2013) | - Substance recovery-related podcast | *Source:* Pre-existing podcast  *Presenters:* Not reported | *Theory:* Alcoholics Anonymous 12-step program  *Engagement:* Professional addiction counsellors reviewed content appropriateness. | *Number:* Not reported  *Frequency:* Not reported  *Duration:* Not reported (Total of ~48 hours of content was available) | Recovery-related content |
| Shaygan (2021) | - Intervention: Online multimedia psychoeducational condition | *Source:* Not reported  *Presenters:* Not reported | *Theory:* Cognitive Behaviour Therapy (Beck, 2011); Mindfulness Based Stress Reduction (Kabat-Zinn, 2013);  Relaxation techniques; Positive Psychotherapy  *Engagement:* Not reported | *Number:* Not reported  *Frequency:* Not reported  *Duration:* Not reported | Not reported |
| Stork (2019) | - Control condition | *Source:* Pre-existing podcast  *Presenters:* Not reported | *Theory:* Not reported  *Engagement:* Not reported | *Number:* 1  *Frequency:* Once off  *Duration:* 7 minutes | History of consumerism |
| Tavakolizadeh (2021) | - Intervention case condition | *Source:* Not reported  *Presenters:* Not reported | *Theory:* Not reported  *Engagement:* Not reported | *Number:* 8  *Frequency:* Weekly  *Duration:* 90 minutes | Wellbeing topics including relaxation, meditation and forgiveness |
| Turner-McGrievy (2009) | - Intervention enhanced podcast condition | *Source:* Researcher developed podcast  *Presenters:* Not reported | *Theory:* Social Cognitive Theory; User Control Theory; Cognitive Load Theory; Elaboration Likelihood Model  *Engagement:* Not reported | *Number:* 24  *Frequency:* Twice weekly  *Duration:* 15:42 minutes | Behavioural strategies to improve nutrition and physical activity behaviours with a focus on achieving a healthy weight and goal setting. |
|  | - Control condition | *Source:* Pre-existing podcast  *Presenters:* Two presenters | *Theory:* Not reported  *Engagement:* Not reported | *Number:* 24  *Frequency:* Twice weekly  *Duration:* 18:34 minutes | How to lose weight and cognitive restructuring to avoid overeating to achieve a healthy weight. |
| Turner-McGrievy (2011) | - Podcast + social media intervention condition - Podcast control condition | *Source:* Researcher developed podcast  *Presenters:* Not reported | *Theory:* Social Cognitive Theory  *Engagement:* Not reported | *Number:* 48 (24 podcasts, 24 mini-podcasts)  *Frequency:* Twice weekly  *Duration:*   - Podcasts 15:42 minutes - Mini-podcasts 5 minutes | Podcasts (0-3 months): Nutrition, physical activity information, weight loss, goal setting.  Mini-podcasts (3-6 months): Nutrition, exercise, overcoming barriers and problem-solving issues. |
| Turner-McGrievy (2017) | - Bite Counter condition | *Source:* Researcher developed podcast  *Presenters:* Not reported | *Theory:* User Control Theory; Cognitive Load Theory  *Engagement:* Not reported | *Number:* 8  *Frequency:* Twice weekly  *Duration:* 15 minutes | Information related to diet, goal setting; Planning and using the Bite Counter at social gatherings |
| Turner-McGrievy (2017) | - App condition - Bite condition | *Source:* Researcher developed podcast  *Presenters:* Not reported | *Theory:* Social Cognitive Theory; Diabetes Prevention Program  *Engagement:* Not reported | *Number:* 48 (24 podcasts, 24 mini-podcasts)  *Frequency:* Twice weekly  *Duration:*   - Podcasts 15:42 minutes - Mini-podcasts 5 minutes | Podcasts (0-3 months): Nutrition, physical activity information, weight loss, goal setting.  Mini podcasts (3-6 months): Nutrition, exercise, overcoming barriers and problem-solving issues. |
| Wahbeh (2016) | - Control/Comparator: Podcast + other was used in Internet education condition | *Source:* Pre-existing podcast.  *Presenters:* Not reported | *Theory:* Mindfulness Based Cognitive Therapy; Mindfulness Based Stress Reduction  *Engagement:* Not reported | *Number:* 6  *Frequency:* Weekly  *Duration:* Not reported | Healthy Eating; Healthy Exercise; Healthy Sleep; Healthy Brain; Healthy Mood; Community Involvement |
| **Grey literature – PhD Dissertation and Theses** | | | | | |
| Dahl (2013) | - Intervention condition: Healthy Eating and Physical Activity | *Source:* Researcher developed *Presenters:* Not reported | *Theory:* Social Cognitive Theory  *Engagement:* Not reported | *Number:* 10  *Frequency:* All available at once  *Duration:* 21:47 minutes | Healthy Eating and Physical Activity during pregnancy; lifestyle and behavioural strategies; and postpartum advice. |
|  | - Control condition: Stress Reduction and Management | *Source:* Pre-existing podcast  *Presenters:* Not reported | *Theory:* Not reported  *Engagement:* Not reported | *Number:* 10  *Frequency:* All available at once  *Duration:* 10:56 minutes | Stress reduction, behavioural and mediation |
| Duffy (2013) | - Intervention condition | *Source:* Not reported  *Presenters:* Not reported | *Theory:* Not reported  *Engagement:* Not reported | *Number:* Not reported  *Frequency:* Not reported  *Duration:* Not reported | Not reported |
| Dunston (2020) | - Intervention condition | *Source*: Pre-existing podcast  *Presenters:* Not reported | *Theory:* Plan-Do-Study-Act model  *Engagement:* Not reported | *Number:* 3  *Frequency:* All available at once  *Duration:* ~10-20 minutes | Positive coping and behavioural strategies, nutritional and physical activity information. |
| Kazen (2018) | - Intervention condition | *Source:* Researcher developed podcast  *Presenters:* One presenter | *Theory:* Brief Interpersonal Psychotherapy  *Engagement:* Not reported | *Number:* 6  *Frequency:* every 3-days for 3-weeks  *Duration:* 10:30 minutes | Relationship separation, moving on and exploring self-concept and self-esteem. |
| Nkwocha (2022) | - Intervention condition | *Source:* Researcher developed  *Presenters:* Not reported | *Theory:* Social Cognitive Theory  *Engagement:* Not reported | *Number:* Not reported  *Frequency:* Twice weekly  *Duration:* Not reported | Reducing risks of cardiovascular disease; type 2 diabetes mellitus, obesity, and overweight |
| **Grey Literature – Published Protocol’s** | | | | | |
| Huberty (2022) | - Health education podcast app (control) condition | *Source:* Not reported  *Presenters:* Not reported | *Theory:* Not reported  *Engagement:* Not reported | *Number:* 20  *Frequency:* Weeks 1-5 uploaded weekly. Weeks 5-20 remaining episodes all available from Week 5.  *Duration:* Minimum listen time of 10 minutes per day | Topics that excludes any recommendations for sleep, meditation, or mindfulness-like principles or practice. |
| Turner McGrievy (2020) | - Intervention condition: whole foods, plant-based vegan diet. - Intervention condition: low-fat omnivorous diet. | *Source:* Researcher developed podcast  *Presenters:* Not reported | *Theory:* Social Cognitive Theory  *Engagement:* Not reported | *Number:* Not reported  *Frequency:* Twice monthly  *Duration:* Not reported | Topics focus on behavioural strategies for dietary maintenance |
| Woodworth (2023) | - Podcast control condition | *Source:* Pre-existing podcast  *Presenters:* Not reported | *Theory:* Not reported  *Engagement:* Not reported | *Number:* 69  *Frequency:* Not reported  *Duration:* Minimum listen time of 10 minutes per day | Knowledge-based topics related to managing needs of caregivers. |
| **Grey literature – Clinical Trial Registers** | | | | | |
| Arnold (n.d.) | - Intervention condition: Education | *Source:* Pre-existing  *Presenters:* Three or more presenters | *Theory:* Social Cognitive Theory; Self-efficacy Theory; Self-determination Theory; Transtheoretical Model  *Engagement:* Not reported | *Number:* 2  *Frequency:* Once every five weeks  *Duration:* 52.50 minutes | The Academy of Imperfection – Ben Crowe; How to progress your running sensibly. |
| Fitzpatrick (n.d.) | - Intervention condition | *Source:* Not reported  *Presenters:* Not reported | *Theory:* Not reported  *Engagement:* Not reported | *Number:* Not reported  *Frequency:* Not reported  *Duration:* Not reported | Not reported |
| Hammer (n.d.) | - Intervention condition | *Source:* Not reported  *Presenters:* Not reported | *Theory:* Not reported  *Engagement:* Not reported | *Number:* Not reported  *Frequency:* Not reported  *Duration:* Not reported | Not reported |
| Jácome-Hortua (n.d.) | - Intervention condition | *Source:* Not reported  *Presenters:* Not reported | *Theory:* Not reported  *Engagement:* Not reported | *Number:* Not reported  *Frequency:* Not reported  *Duration:* Not reported | Nutritional recommendations |
| Janevic (n.d.) | - Intervention condition | *Source:* Researcher developed  *Presenters:* three or more presenters | *Theory:* Not reported  *Engagement:* Community advisory board | *Number:* 6  *Frequency:* Weekly  *Duration:* 30 minutes | Information related to physical activity, diet/nutrition, wellbeing and selfcare. |
| Klein (n.d.) | - Intervention condition | *Source:* Not reported  *Presenters:* Not reported | *Theory:* Not reported  *Engagement:* Not reported | *Number:* Not reported  *Frequency:* Not reported  *Duration:* Not reported | Not reported |
| Mikocka-Walus (n.d.) | - Intervention condition | *Source:* Not reported  *Presenters:* Not reported | *Theory:* Not reported  *Engagement:* Not reported | *Number:* Not reported  *Frequency:* Not reported  *Duration:* Not reported | Not reported |
| Phillips (n.d.) | - Intervention condition Buddy | *Source:* Not reported  *Presenters:* Not reported | *Theory:* Not reported  *Engagement:* Not reported ported | *Number:* 4  *Frequency:* Once every three weeks  *Duration:* 10-15 minutes | Not reported |
| Phillips (n.d.) | - Intervention condition: Buddy | *Source:* Not reported  *Presenters:* Not reported | *Theory:* Not reported  *Engagement:* Not reported | *Number:* 4  *Frequency:* Once every three weeks  *Duration:* 10-15 minutes | Not reported |
| Pirzadeh (n.d.) | - Intervention condition: Education | *Source:* Not reported  *Presenters:* Not reported | *Theory:* Not reported  *Engagement:* Not reported | *Number:* Not reported  *Frequency:* Not reported  *Duration:* Not reported | Not reported |
| Pirzadeh (n.d.) | - Intervention condition | *Source:* Not reported  *Presenters:* Not reported | *Theory:* Not reported  *Engagement:* Not reported | *Number:* Not reported  *Frequency:* Not reported  *Duration:* Not reported | Not reported |
| Amirzadegan (n.d.) | - Intervention condition | *Source:* Researcher developed  *Presenters:* Not reported | *Theory:* Not reported  *Engagement:* Not reported | *Number:* Not reported  *Frequency:* Not reported  *Duration:* 20-30 minutes | COVID-19 self-care tutorials |
| Rio (n.d.) | - Intervention condition | *Source:* Not reported  *Presenters:* Not reported | *Theory:* Not reported  *Engagement:* Not reported | *Number:* Not reported  *Frequency:* Not reported  *Duration:* Not reported | Not reported |
| Turner-McGreivy (n.d.) | - Intervention condition - Control condition | *Source:* Researcher developed  *Presenters:* Not reported | *Theory:* Not reported  *Engagement:* Not reported | *Number:* Not reported  *Frequency:* Twice weekly  *Duration:* Not reported | Not reported |
| Weisel (n.d.) | - Intervention condition | *Source:* Not reported  *Presenters:* Not reported | *Theory:* Acceptance and commitment therapy  *Engagement:* Not reported | *Number:* 4  *Frequency:* Weekly  *Duration:* Not reported | ACT values and needs-based exercises. |
| **Grey literature – Conference Abstracts** | | | | | |
| Peaceman (2017) | - Intervention condition | *Source:* Not reported  *Presenters:* Not reported | *Theory:* Not reported  *Engagement:* Not reported | *Number:* Not reported  *Frequency:* Not reported  *Duration:* Not reported | Not reported |
| Weisbrod (2019) | - Intervention condition | *Source:* Not reported  *Presenters:* Not reported | *Theory:* Not reported  *Engagement:* Not reported | *Number:* Not reported  *Frequency:* Not reported  *Duration:* Not reported | Not reported |

Key:

^a^ Information for this study was collated from multiple papers relating to the same study.
