# Supplementary material for: Podcasts in Mental, Physical, or Combined Health Interventions for Adults: Scoping Review
Source: J Med Internet Res. 2025 May 7;27:e63360. doi: 10.2196/63360 (PMC12096026; doi:10.2196/63360)
Supplement: Multimedia Appendix 7 [file jmir_v27i1e63360_app7.docx]

**Supplementary Table 3: Podcast usage data process evaluation data and related information**

| **Author (year)** | **Podcast usage data^b^** | **Podcast facilitators^c^** | **Podcast barriers^d^** | **Other perceptions of podcast use** |
| --- | --- | --- | --- | --- |
| **Peer reviewed articles** | | | | |
| Anderson (2017) | - Not reported | - Not reported | - Not reported | - Not reported |
| Bangia (2014) | - Not reported | - *Practical:* Easy dissemination of information - *Accessibility:* Free-to-use - *Format:* Brevity of podcast, clarity of information | - *Technical:* Improving the podcast's sound clarity. - *Format:* Some noted podcasts as “tedious” and “confusing” as podcast information did not align with layout of shop aisles. | - 89% (*n* = 50) of participants liked the podcast |
| Bangia (2017) | - Not reported | - Not reported | - Not reported | - Not reported |
| Cai (2023) | Number of podcasts used % (*n*/78):   - 0: 18% (*n* =14) - 1-2: 18% (*n* =14) - 3-4: 9% (*n* =7) - 5-7: 55.1% (*n* =43)   Time spent listening to podcast % (*n*):   - 0-10 minutes: 9.4% (*n* = 6) - 11-20 minutes: 4.7% (*n* = 3) - 21-30 minutes: 7.8% (*n* = 5) - 31-60 minutes: 17.2% (*n* = 11) - > 1 hour: 60.9% (*n* = 39)   Overall, 82.1% (*n* = 64) listened to at least 1 podcast. | - *Accessibility:* access on demand, podcast scripts were available - *Format:* Script underwent readability tests - *Brevity:* Episode length: 10 to 20 minutes was chosen to keep listeners engaged and to allow for podcasts to fit into patient lifestyles | - Not reported | - 97% would recommend podcast. - Increased patient satisfaction:   Rating of podcast for labour education:   - - Mean (SD): 8.7 (1.4)   (1 terrible to 10 excellent) |
| Davies (2021) | Podcasts were queued for participants to listen. Podcasts were played only once. | - Not reported | - Not reported | - Not reported |
| Davies (2022) | Podcasts were queued for participants to listen. Podcasts were played only once. | - Not reported | - Not reported | - Not reported |
| Dunn (2019) | Total number (out of 48) of podcasts downloaded Mean (SE):   - Photo condition (*n* = 22): 14.2 (13) - Calorie condition (*n* =19): 15.0 (13.9) | - *Technical:* Podcast engagement used objective measures of tracking frequency and podcasts downloaded. - *Accessibility:* Researchers helped participants at orientation with navigation and technical difficulties and were available by telephone or e-mail throughout the study. | - Not reported | - Not reported |
| Edwards (2021) & Shaw (2022)^a^ | All participants self-reported listening to podcasts episodes 1-5, with ~50% listening to episode 6 (optional). | - *Format:* Content was relatable and representative. Shared stories were honest, authentic, and engaging. Enhanced knowledge and understanding of menopause. Podcasts reframed menopause (reduced “taboo” /stigma), stimulated conversations. | - *Format:* Podcast content was unrelatable and not representative (e.g., some shared stories). | - Participants reported being motivated to implement lifestyle changes to improve physical and mental health. |
| Hales (2016) | Theory-based podcast downloads did not significantly differ between groups, Mean, SD and 95% (CI): *p* = 0.22   - Social Pounds Off Digitally app + theory-based podcast: 20.9 ± 13.5 (95% CI: 14.9, 26.9) - Control app + theory-based podcast: 16.5 ± 10.3 (95% CI: 12.2, 20.8) | - Not reported | - *Accessibility:* The Social Pounds Off Digitally app was only available for the Android operating system (excluding other smartphone and non-smartphone users). | - Not reported |
| Huberty (2020) | Feasibility benchmarks:   - Podcasts listened to, Mean (SD):   - 103.2 (29.5) minutes per week.   - 4.9(0.9) podcast sessions each week - Demand 83.3% (10/12) of the weeks at least 70% of participants completed prescribed listenership. - Acceptability benchmarks were not met:   - Enjoyment: 48.7% (*n* = 19)   - Satisfaction 43.6% (*n* = 17) | - *Format:* learned new information (*n* = 21, 53.8%) | Overall, 51.3% (*n* =20) participants reported experiencing some form of barrier.   - *Accessibility:* Difficulty hearing /viewing podcast 10.3% (*n* = 4); app functionality 46.7% (*n* =7); accessing specific podcasts 33.3% (*n* =5); accessing any app content 20% (*n* =3). - *Technical:* internet connection 2.6% (*n* = 1) - *Format:* 62% *(n* = 21) changes to podcast content (increased relatability or did not enjoy certain podcasts). | - 56.4% (*n* = 22) would recommend podcast. - Implemented lifestyle changes due to podcast information 51.3% (*n* = 20) - *Attrition:* considered as qualifier in future podcast engagement. |
| Kanstrup (2021) | - All participants in the control condition listened to the podcast. | - Not reported | - Not reported | - Not reported |
| Laird (2022) | - Not reported | - Not reported | - Not reported | - Not reported |
| Lui (2021), Lui (2022) & Wilcox (2022)^a^ | Podcast downloads pregnancy, Mean (SD):   - Behavioural intervention: 7.2 (3.7) - Standard care: 6.4(4.2)   Podcast downloads postpartum:   - Behavioural intervention: 5.2 (6.2) - Standard care: 6.2 (6.8) | - *Accessibility:* Accessing information was easy, Mean (SD):   - Pregnancy: 6.7 (0.7)   - Postpartum: 6.4 (1.0)   (1 totally disagree to 7 completely agree)   - *Format:* effective way to get information about healthy eating, Mean (SD):   - Pregnancy: 5.8 (1.6)   - Postpartum: 5.1 (1.8)   (1 totally disagree to 7 completely agree)   - *Format:* effective way to get information about weight control / weight loss, Mean (SD):   - Pregnancy: 5.5 (1.7)   - Postpartum: 5.0 (1.8)   (1 totally disagree to 7 completely agree)   - *Format:* effective way to get information about exercise, Mean (SD):   - Pregnancy: 5.5 (1.6)   - Postpartum: 5.0 (1.8)   (1 totally disagree to 7 completely agree) | - *Accessibility:* Some participants did not have a device that allowed them to access the podcast, the study sent a CD with that week’s podcast on it. - *Technical:* Researchers were unable to measure podcast listening time. - *Format:* Content reiterated the same covered in other intervention components. Duration of all podcasts was too long, and frequency of postpartum podcasts too many. - *Attrition:* Number of postpartum podcasts downloads was low. | - Overall podcast quality, Mean (SD):   - Pregnancy: 2.9 (1.0)   - Postpartum: 2.6 (0.9)   (0 poor to 3 excellent)   - Podcast helpfulness, Mean (SD):   - Pregnancy: 2.0 (0.8)   - Postpartum: 1.9 (0.6)   (0 did not help at all to 3 helped a lot)   - Number of podcasts (%):   - Pregnancy     - Too few: 3.5%     - Just about right: 70.9%     - Too many: 25.6%   - Postpartum     - Too few 0%     - Just about right: 37.8%     - Too many: 62.2% |
| Mailey (2016) | - >85% of participants who completed the program evaluation at week 8 (*n* = 37) reported listening to the podcasts during weeks 1 and 2. - 55% reported listening to the podcasts during weeks 7 and 8. | - *Innovative* - *Format:* Brevity, short and succinct | - *Format:* Duration and concerns about content appropriateness | - Podcasts were reported as best intervention component (*n* = 19) and least favourite aspects (*n* = 7). |
| Mailey (2019) | - Not reported | - *Innovative:* Podcasts were novel - *Accessibility:* “podcasts was convenient”, “could listen to them while doing other activities”. - *Format:* Good range of topics | - *Accessibility:* “I had difficulties getting to the podcasts and gave up” - *Format:* “too long” duration | - Overall podcast rating: 6.20   (Range 1 to 10)   - *Format:* General dislike “I don't like podcasts (trying to listen to these was tedious)” |
| Militello (2021) | App usage (self-report after listening to each podcast episode). Plus, exit interviews at 2-weeks.  Proportion of podcasts listens by category (*N* = 239), *n* (%):   - Sleep *n* = 81 (33.9%) - Pregnancy *n* = 72 (30.1%) - Parenting *n* = 72 (30.1%) - Breastfeeding *n* = 36 (15.1%) | - *Format:* Desire content that is sensible, objective, balanced between old and new information. | - *Technical:* Application system failed to capture data accurately. Participants would like podcasts available on other devices (e.g., iPhone or Android) - *Format:* Podcasts were not practical or relevant. - *Attrition:* Number of users per episode was reduced by nearly half during episode 15. Reasons unclear. | - Not reported |
| Seib (2022) | - Not reported | - Not reported | - Not reported | - Not reported |
| Shaw (2013) | - 56% reported using the iPod for ≥6 days. - 67% reported using the iPod for ≥20 minutes per day. | - *Format:* Useful, would like access podcast recovery resources sooner to aid recovery - *Accessibility:* can assess at various locations | - *Accessibility:* despite issuing user guide, not all users had sound technical skills. iPods usability improved as study progressed indicating intervention was easy to learn. | Participant feedback, Mean:   - Comfort level with podcasts: 2.1 - How relevant was information: 4.5 - Would like access to an iPod like one used during study: 4.5 - How useful was the iPod to use in different locations?   - On the move: 4.36   - At home: 4.27   - When in bed: 3.57   - While exercising: 3.54   - At work: 2.69   (1 Not at all to 5 Very much so) |
| Shaygan (2021) | - Not reported | - Not reported | - Not reported | - Not reported |
| Stork (2019) |  |  |  | Music condition was superior to the podcast control condition, Mean:   - Liking:   - - Music 7.83     - Podcast 5.67   (0 I did not like it at all to 10 I liked it very much)   - Motivation   - Music: 8.04   - Podcast 3.21   (0 it did not motivate me at all to 10 it motivated me very much) |
| Tavakolizadeh (2021) | - Not reported | - Not reported | - Not reported | - Not reported |
| Turner-McGrievy (2009) | Podcasts listened to out of 24 episodes, Mean (SD):   - Intervention condition: 17.5 (8.1) - Control condition: 16.6 (7.5) | - *Accessibility:* Low-cost, easy dissemination - *Format:* The enhanced podcast weekly soap opera format was an entertaining way to provide healthy eating and exercise information. | - *Format:* control podcast format varied among episodes. | - Locations podcasts were listened to:   - 53.7% at home   - 20.9% office/ work,   - 13.4% walking or exercising. - Activity while listening:   - 44.8% sitting at desk,   - 22.4% sitting at home. - *Device type:* 52.2% listened to podcasts on their computer versus portable MP3 player. - *Listening habits:* 85% did not listen to any other weight-loss or health-related podcasts during the study. |
| Turner-McGrievy (2011) | Podcast + social media intervention condition:   - Podcast downloads (out of 24; self-report), Mean (SD):   - 0-3 months: 16.4 (7.2)   - 3-6 months: 9.0 (9.1) - Number of downloads per podcast episode per person from host site*:   - 0-3 months: 2.0 (0.5)   - 3-6 months: 0.9 (0.2), - Number of total podcast downloads from podcasting host site*:   - 0-3 months: 94.1 (24.6)   - 3-6 months: 40.7 (9.5)   Podcast control condition:   - Podcast downloads (out of 24; self-report), Mean (SD):   - 0-3 months: 14.5 (7.6)   - 3-6 months: 8.2 (8.6) - Number of downloads per podcast episode per person from host site, Mean (SD)*:   - 0-3 months: 1.5 (0.7)   - 3-6 months: 0.7 (0.2) - Number of total podcast downloads from podcasting host site, Mean (SD)*:   - 0-3 months: 74.2 (31.8)   - 3-6 months: 32.5 (7.2)   * Significant difference between Podcast + social media intervention group and Podcast control group (*p*<.001) | - Not reported | - Not reported | - Not reported |
| Turner-McGrievy (2017) | Podcast downloads out of two per week, Mean (SE):   - Week 1: 1.6 (0.2) - Week 2: 1.1 (0.3) - Week 3: 1.2 (0.3) - Week 4: 1.1 (0.3) | - Not reported | - Not reported | - Not reported |
| Turner-Mcgrievy (2017) | Podcast downloads out of 48, Mean (SE):   - Standard app condition (*n* = 42): 31.0 (2.7) - Bite condition (*n* = 39): 26.1 (2.8)   * There were no differences in total number of podcasts downloaded between groups. | - *Accessibility:* User guide / instructions issued at baseline assessment, and received a test podcast to ensure mobile device could download and play the files - *Technical:* Podcast links were emailed twice weekly to participants (allowing objective assessment of utilization) | - *Technical:* user errors and software issues. | - Podcasts downloaded appeared to be important for weight loss, regardless of group assignment.   * Total weight loss was significantly and moderately correlated with the number of podcasts downloaded (*r* -0.33, *p* < 0.01). |
| Wahbeh (2016) | Control internet education condition, Mean (SD), Range:   - Sessions completed: 4.75 (1.8), Range = 2-6 - Home practice minutes: 873 (395) minutes, Range = 327-1524 - Days of practice: 25.6 days, Range = 11-35 | - *Accessibility /Technical:* Researchers switched from iPads to participants own desktop or laptop computers to access the online programs and installing iMindr on an iPod Touch. This combination was more amenable to the participants. | - *Accessibility / Technical:* Researchers found that iPads were difficult to use for many of the participants. | - Not reported |
| **Grey Literature – PhD Dissertations and Theses** | | | | |
| Dahl (2013) | - Not reported | - Not reported | - Not reported | - Primigravida women expressed significantly greater interest in podcasts than multiparous women in receiving (80.3% vs. 66.7%, respectively, *p*<0.01) |
| Duffy (2013) | - Not reported | - Not reported | - Not reported | - Not reported |
| Dunston (2020) | Podcasts listenership, % (*N* = 36)   - Episode 1 66.6% (*n* = 24) - Episode 2 16.6% (*n* = 6) - Episode 3 16.6% (*n* = 6) |  | - *Technical:* podcast platform released all podcast simultaneously, rather than on separate occasions as planned. | - No post-intervention questions were included to assess podcast utility. |
| Kazen (2018) | Podcast listenership received by *n* = 11:   - 64% (*n* = 7) listened all podcasts - 18% (*n* = 2) listened 5 episodes - 9% (*n* = 1) listened 3 episodes - 9% (*n* = 1) listened 0 | - *Accessibility:* Ease of access - *Format:* Useful, appropriate content, easy to follow/understand. | - *Accessibility:* Waiting for podcasts - *Format*: Limited number of podcasts - *Format:* some content was not relatable to individual’s situation. Presenters tone and voice were “too cheerful” for the subject matter. Must listen to all podcasts to get full benefit. | - 80% would recommend program to others. - Listening location:   - At home 91% (*n* = 10)   - Outside home 9% (*n* = 1) - Device:   - Smart phone 91% (*n* = 10)   - iPad 9% (*n* = 1) |
| Nkwocha (2022) | - Not reported | - Not reported | - Not reported | - Not reported |

Key:

^a^ Information for this study was collated from multiple papers relating to the same study.

^b^ Usage data (quantitative if reported, or qualitative if not specifically reported but mentioned in article)?

^c^ Facilitators to use and implementation (described as ‘*a person or thing which facilitates action, process, results’* e.g., something that aids, helps, enhances engage with podcast/s)?

^d^ Barriers to use and implementation (described as ‘*any natural obstacle which stops or obstructs passage… prevents access’* in relation to podcast/s engagement)?
